# Comprehensive health assessment of retired martial arts athletes: bone density, dietary intake, physical activity, and wellbeing

**DOI:** 10.3389/fragi.2025.1513936

**Published:** 2025-02-03

**Authors:** Tasneem Alshaer, Nihad Battikhi, Adam Tawfiq Amawi, Khalid Trabelsi, Haitham Jahrami, Philippe Bouedo, Hadeel Ali Ghazzawi

**Affiliations:** ^1^ Department of Nutrition and Food Technology, School of Agriculture, The University of Jordan, Amman, Jordan; ^2^ School of Sport Sciences, Department of Movement Sciences and Sports Training, The University of Jordan, Amman, Jordan; ^3^ High Institute of Sport and Physical Education of Sfax, University of Sfax, Sfax, Tunisia; ^4^ Research Laboratory: Education, Motricity, Sport and Health, EM2S, LR19JS01, University of Sfax, Sfax, Tunisia; ^5^ Department of Rehabilitation Medicine Services, Government Hospitals, Manama, Bahrain; ^6^ Department of Psychiatry, College of Medicine and Health Sciences, Arabian Gulf University, Manama, Bahrain; ^7^ Chair of the Technical Commission (the World Taekwondo Games), , Seoul, South Korea

**Keywords:** bone density, bone mineral content, weight, martial arts, retired athletes, former athletes, sports, weight-dependent sports

## Abstract

Regular physical activity offers benefits like enhanced bone density, which often persists post-retirement. However, retirement can lead to weight gain and changes in quality of life due to reduced physical activity. Therefore, this study investigates the long-term effects of elite athletic experience by comparing retired Jordanian male elite athletes (ages 40–50) with non-athletes of the same age. The differences were explored in weight changes, bone density, quality of life, happiness, stress, insomnia, physical activity, and dietary intake to understand how martial arts impact these health dimensions.

**Methods:**

A descriptive case-control study was conducted among 30 retired male elite athletes and 20 age-matched non-athletes. Bone density and body composition were measured using dual-energy X-ray absorptiometry (DEXA) scans. Additional assessments included anthropometric measurements, a 3-day dietary recall, physical activity (International Physical Activity Questionnaire), quality of life (WHOQOL), happiness (Subjective Happiness Scale), stress (Perceived Stress Scale), and insomnia (Insomnia Severity Index).

**Results:**

Retired athletes showed significantly higher Z-scores for the left femur (neck and total) and the AP spine, with *p*-values <0.05. Among non-athletes, 65% had normal bone density with a Z-score ≥ −1.9 and a T-score > −1.1, 20% had abnormal bone density with a Z-score < −1.9, and 15% had osteopenia with a T-score between −1.1 and −2.4. In contrast, 100% of retired athletes had normal bone density with a Z-score ≥ −1.9 and a T-score > −1.1. Retired athletes exhibited greater weight changes than non-athletes, with a *p*-value <0.05; the average weight gain among retired athletes was 18.548 kg, and the mean weight gain among non-athletes was 4.3 kg. There were statistically significant mean differences in perceived stress levels between retired athletes and non-athletes with a *p*-value <0.05. In contrast, there were no statistically significant mean differences between the groups in quality of life, subjective happiness, and the Insomnia Severity Index.

**Conclusion:**

The study reveals that retired elite athletes maintain better bone density but face greater weight gain and stress than their non-athlete counterparts. Both groups enjoy a high quality of life and low levels of insomnia. These findings underscore the importance of continued physical activity for health and suggest that both retired athletes and non-athletes should adopt a balanced lifestyle to manage weight and stress effectively.

## 1 Introduction

Elite athletes are individuals who have reached the peak of their sport, demonstrating exceptional performance and dedication at a competitive level (McAuley et al., 2020). Regular physical activity is widely recognized as an integral component of a healthy lifestyle, offering numerous benefits for physical and mental wellbeing (Adamson et al., 2022; World Health Organization, 2018). Among the various forms of physical activity, martial arts (MA) stand out for their unique combination of health benefits, including improvements in physical fitness, social skills, and self-confidence (Cynarski, 2018; Origua Rios et al., 2018).

Research indicates that physical activity is recognized as an integral component of a healthy lifestyle, providing multiple health and wellbeing advantages as individuals age. It helps prevent diseases such as stroke, heart disease, diabetes, and osteoporosis (Adamson et al., 2022; World Health Organization, 2018). In addition, it plays a significant role in promoting bone health, irrespective of gender (Kopiczko et al., 2021). Achieving peak bone mass through regular exercise is essential for decreasing bone loss associated with aging and protecting both bone and muscle tissues from deterioration (Küçükkubaş and Korkusuz, 2020). Notably, studies have shown that even many years after retiring from sports, athletes still have stronger bones than sedentary individuals (Eser et al., 2009).

Despite the recognized benefits of athletic training during an athlete’s career, the transition to retirement often presents a range of health challenges. Retired athletes are at an increased risk of various physical and mental health issues, including musculoskeletal injuries, chronic pain, and psychological concerns such as depression and anxiety (Voorheis et al., 2023; Reardon et al., 2019).

In addition, it affects an athlete’s quality of life, presenting both challenges and opportunities. On the other hand, the sudden change in routine and the loss of a clearly defined purpose can lead to feelings of uncertainty, sadness, stress, and even depression (Melekoğlu et al., 2019; Sanders and Stevinson, 2017), particularly for athletes who are unprepared for the transition. Uncertainty about the future, financial concerns, and the need to adjust to a new, less structured routine are some contributing factors (Hatamleh and Mazin, 2013). Moreover, retired athletes frequently experience insomnia, which is linked to psychological distress and disrupted routines during this transitional phase (Fernandes et al., 2019).

In addition, many retired athletes follow sedentary and unhealthy lifestyles characterized by reduced physical activity and poor dietary habits (Yao et al., 2020). These lifestyle changes can lead to alterations in body composition, such as weight gain and muscle loss, leading to long-term health complications and reduced quality of life, including metabolic syndrome and cardiovascular diseases (Voorheis et al., 2023; Emami et al., 2018).

These interconnected physical and psychological factors highlight the importance of studying health variables such as bone density, body mass index (BMI), quality of life, stress, and insomnia in retired athletes as they highlight the urgency of providing targeted interventions and lifestyle recommendations to support retired athletes in maintaining their health and quality of life. Several studies tend to focus on active athletes; there is a noticeable lack of research on the experiences and health outcomes of retired athletes, such as changes in body weight and bone density, and recommendations for their lifestyle, particularly in the Middle East, where studies are still limited. This study investigates the long-term effects of elite athletic experience by comparing retired Jordanian male elite athletes (ages 40–50) with non-athletes of the same age. The differences in weight changes, bone density, quality of life, happiness, stress, insomnia, physical activity, and dietary intake were explored to understand how martial arts impact these health dimensions.

## 2 Materials and methods

### 2.1 Research design

A descriptive case-control study assessed the body weight changes and bone density among retired Jordanian male elite martial arts athletes.

A convenience sample of 30 healthy retired Jordanian male elite martial arts athletes (taekwondo, boxing, judo, karate, and kickboxing), aged 40–50, who have not participated in any competitions in the last 10 years, and 20 healthy non-athlete Jordanian male individuals, aged 40–50, were included in this study. The sample size was limited due to constrained research funds and the fact that this is a preliminary study.

**FIGURE 1:**
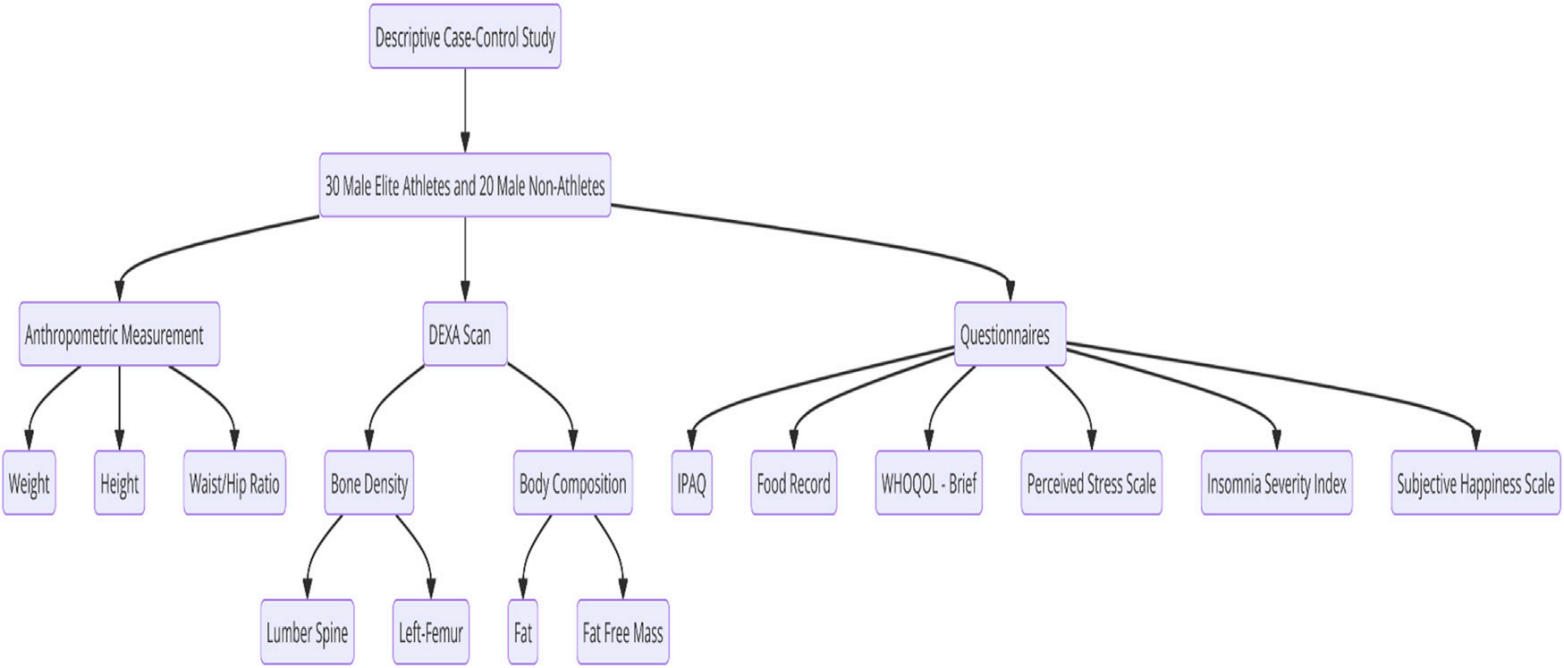
Flow diagram of the study design.

For athletes, 43 individuals filled out the participation form, but 30 fit the exclusion and inclusion criteria. For non-athletes, 51 filled out the participation form, but 20 fit the exclusion and inclusion criteria. All participants were recruited from Jordan, and the athletes were recruited from the Jordanian martial arts federations. Recruited participants were interviewed and informed briefly about the benefits and objectives of the study; an informed consent form was obtained from each participant before enrollment in the study; and socio-demographic data were collected. Figure 1 shows the flow diagram of the study design.

We excluded individuals who were female or active elite athletes and athletes who had been retired for less than 10 years. Additional exclusion criteria included being underage or overage, using drugs that affect body composition, having any chronic disease, experiencing a bone injury or fracture within the past 6 months, having any bone disease or immune disorder, taking medications that affect bone density, or using vitamin D supplements.

### 2.2 Data collection

#### 2.2.1 Anthropometric measurement

Basic anthropometric measurements, including body weight, height, waist and hip circumference, and the waist–hip ratio, were obtained from each participant. For athletes, actual body weight was compared with the weight category they used to compete in, while for non-athletes, actual body weight was compared with their weight from 10 years ago.

#### 2.2.2 Medical measurement

##### 2.2.2.1 Dual-energy X-ray absorptiometry

The dual-energy X-ray absorptiometry (DEXA) scan was conducted using Lunar iDXA-GE (Madison, WI, United States) at the National Center for Diabetes, Endocrinology, and Genetics (NCDEG) to determine bone density and estimate whole-body composition, including fat and fat-free mass. The participants visited the center only once at a convenient time for the examination, which took approximately half an hour under the supervision of a specialized X-ray technician. The reliability of the DEXA scan was validated (intraclass correlation coefficient (ICC) ≥ 0.95) by Moreira et al. (2018) for segmental body composition measuring.

The DEXA scan is rapidly becoming more accessible and popular as a technique. It provides estimations by measuring the body’s absorbance of X-rays at two different energies, given that fat, bone mineral, and fat-free soft tissue have varied absorption properties. According to the manufacturer’s protocol, the participants were positioned for whole-body scans (Peppa et al., 2017; Nana et al., 2015).

Bone measurements included the lumbar spine (i.e., L1 to L4) and hip area (left femur: neck, Wards, troch, shaft, and total); the results were based on Z-scores for men aged <50 and T-scores for men aged ≥50. For the lumbar (AP) spine, the results were determined using Z-scores or T-scores for L1–L4 for most participants, except for those who had degenerative changes in one of the vertebrae because this could cause overestimation. In such cases, the affected vertebra was excluded, and the results were computed using the remaining vertebrae. For example, in the case of observed degenerative changes in L4, the results from L1 to L3 were calculated. L4 was excluded, and for the left femur, the results were based on the Z-score or T-score of the neck and total, using the lowest score. The Z-score must be −1.9 or more to be normal; the T-score must be more than −1.1 to be considered normal, −1.1 to −2.4 to be considered osteopenia, and −2.5 or less to be considered osteoporosis. In addition to bone density, whole-body composition analysis provided data on total fat and lean mass and its region (trunk, arms, and legs).

##### 2.2.2.2 Dietary assessment

A 3-day food record was used to determine the participant’s dietary intake. The timing of meals or snacks, beverages, food details, cooking methods, and the amounts consumed were recorded. The records were required 3 days per week, consisting of two weekdays and one weekend day (Yang et al., 2010). It is validated with a reliability of ICC ≤0.78 by Kolar et al. (2005). For data analysis, the quantity of food and beverage intake reported by the participant was converted into metric units (gram/kilogram or mL/litter) or using a unit like a cup/teaspoon or tablespoon. The means of energy and macronutrient intakes were determined using the food processor (ESHA, 2011) for each participant. In addition, the means of each group for each nutrient was determined; the Standard Reference Database was used for the dietary analysis. The means of energy and macronutrient intakes were compared with the recommended values.

##### 2.2.2.3 Assessment of quality of life

The Arabic version of the World Health Organization Quality of Life Questionnaire (WHOQOL)-BREF, validated for reliability (α ≥ 0.7) by Oheari and Awadalla, (2009), consists of 26 questions about the participants’ impression of their health, quality of life, or other aspects of their life in the last 4 weeks. It consists of four domains, namely, psychological, physical health, environmental, and social relationships. The WHOQOL-BREF questionnaire utilizes a five-point ordinal scale to score each individual item, ranging from 1 to 5. Subsequently, these scores are linearly transformed to a 0–100 scale, enabling a more comprehensive representation of the participant’s responses (World Health Organization, 2020). For data analysis, SPSS software version 22 was used; it was analyzed according to a score out of 5; a higher score means a higher quality of life, and questions 3, 4, and 26 are negative.

The Arabic version of the Perceived Stress Scale, validated for reliability (α ≥ 0.74) by Almadi et al. (2012), consists of 14 questions related to participants’ attitudes and thoughts over the previous month. Each question asked them to indicate how often they thought a certain way. Participants rate items using a five-point Likert scale, with responses ranging from 0 (“never”) to 4 (“very often”). The total scores range from 0 to 56, with higher scores indicating higher levels of perceived stress (Almadi et al., 2012). For data analysis, SPSS software version 22 was used; it was analyzed according to the scores out of 4, and questions 4, 5, 6, 7, 9, 10, and 3 are positive.

The Arabic version of the Insomnia Severity Index, validated for reliability (α = 0.84) by Suleiman and Yates. (2011), consists of five questions to assess sleep status and problems and their impact on the ability to perform life functions. Each item in the assessment is rated using a five-point Likert scale, where 0 represents “no problem” and 4 indicates a “very severe problem.” The total score is derived by summing up the ratings, ranging from 0 to 28 (Suleiman and Yates, 2011). For data analysis, it was analyzed according to scores. A higher score means higher perceived stress. The valid Arabic Subjective Happiness Scale (SHS), validated with reliability (α = 0.79) by Moghnie and Kazarian (2012), consists of four questions to assess their happiness level in general; it was analyzed according to scores. The scores range from 1 (not at all) to 7 (completely); a high score means high subjective happiness. The responses were summed up and divided by four; the score must be more than 4 (Moghnie and Kazarian, 2012). Data analysis was analyzed according to scores; question 4 is negative.

##### 2.2.2.4 Assessment of physical activity

The Arabic version of the IPAQ-Short Form, validated for reliability (α < 0.80) by Helou et al. (2018) and Craig et al. (2003), consists of seven questions to assess the sitting time and intensity of physical activity that people did as part of their daily lifestyle. They were considered to estimate the total time spent sitting and physical activity in MET-min/week; METs are metabolic equivalents (International Physical Activity Questionnaire, 2014a).

For data analysis, according to guidelines, category 1, the level of physical activity is the lowest; those people who do not meet conditions for categories 2 or 3 are considered insufficiently active (inactive). Category 2, the minimum level of activity, includes any one of the following three criteria: a) at least 20 min/day for 3 days or more of vigorous activity, b) 30 min/day for 5 days or more of moderate-intensity activity or walking, or c) 5 days or more of any mixture of walking, moderate, or vigorous intensity activities achieving at least 600 MET-min/week. Category 3, health-enhancing physical activity (HEPA), the highest active category, includes individuals who exceed the minimum physical activity recommendations and engage in enough activity for a healthy lifestyle; there are two criteria for classification: a) at least 3 days of vigorous-intensity activity achieving at least 1500 MET-minutes/week or b) 7 or more days of any combination of walking, moderate, or vigorous intensity activities achieving at least 3000 MET-minutes/week (International physical activity Questionnaire, 2014b).

##### 2.2.2.5 Ethical considerations

The Institutional Review Board at the University of Jordan (IRB at UJ) approved the research proposal under decision no. (66. 2023). Informed consent was obtained from all participants prior to their inclusion in the study. In addition, it is maintained under strict confidentiality; all personal information was removed from the data, ensuring that individual identities were not disclosed. The data collected were used exclusively for research purposes, and access to the information was restricted to authorized people only.

### 2.3 Statistical analysis part

Statistical analysis was performed using IBM SPSS software version 22. The values of variables were presented as the mean ± standard deviation (SD). The Kruskal–Wallis test was used to compare the two groups, followed by *post hoc* analysis using the Mann–Whitney U test to allocate the differences between groups in comparing overall differences such as weight, bone density, and physical activity. Spearman’s and Pearson correlation tests were used to detect the correlations between overall variables, such as the correlation between bone density and other variables or the weight between other variables. The *p*-value was significant at p < 0.05.

## 3 Results

A descriptive case-control study was conducted in Amman, Jordan, to assess body weight changes and bone density among retired Jordanian male elite martial arts athletes aged between 40 and 50 years.

### 3.1 General characteristics

The general characteristics of the study sample are shown in Table 1. The mean age of the 20 non-athlete male participants was 46.1 ± 3.07 years, with an age range of 40–50, and the mean age for 30 retired athlete male participants was 44.13 ± 3.4 years, with an age range of 40–50. The mean height for non-athletes was 1.75 ± 0.06 (m), with a range of 1.65–1.86, and the mean height for retired athletes was 1.76 ± 0.06 (m), with a range of 1.65–1.92. The mean weight for non-athletes was 90.3 ± 11.37 kg, with a range of 70.4–117.3 (kg), and the mean weight for retired athletes was 88.03 ± 15.04 kg, with a range of 54.7–125.3.

**TABLE 1:** General characteristics of participants.

Group	Characteristic	Mean ± SD
Non-athletes	Age (years)	46.1 ± 3.07
	Height (meters)	1.75 ± 0.06
	Current weight (kg)	90.3 ± 11.37
	Weight before 10 years (kg)	86.10 ± 21.43
Retired athletes	Age (years)	44.13 ± 3.4
	Height (meters)	1.76 ± 0.06
	Current weight (kg)	88.03 ± 15.02
	Weight in the last competition (kg)	69.48 ± 10.86

*Data were represented as the mean ± standard deviation (SD).

### 3.2 Comparison of current weight, weight before 10 years/weight in the last competition, body mass index, and waist–hip ratio among retired athlete and non-athlete male participants

There is no significant difference in current weight between non-athletes and retired athletes (*p*-value > 0.05). Still, there is a substantial difference between the weight before 10 years for non-athletes and the weight in the last competition for retired athletes with a *p*-value = 0.00. The confidence interval (CI) for this difference was (2.5 ± 2.17 kg), indicating that the observed difference is reliable. The effect size (Cohen’s d) was (0.65), suggesting a medium effect.

The body mass index (BMI) was used to assess the body weight status of male retired athletes and non-athletes in Amman. There is no significant difference in the BMI and waist/hip ratio (*p*-value > 0.05), as shown in Table 2.

**TABLE 2:** Mean of current weight, weight before 10 years for non-athletes/weight in the last competition for retired athletes, BMI values, and waist–hip ratio among the sample.

Variable	Non-athletes N = 20	Retired athletes N = 30	*p*-value
Current weight	90.30 ± 11.37	88.028 ± 15.04	0.47
Weight before 10 years/weight in the last competition	86.10 ± 21.43	69.48 ± 10.86	**> 0.00**
BMI	30 ± 3.4	28.6 ± 4.2	0.22
Waist–hip ratio	1.35 ± 0.11	1.27 ± 0.25	0.17

*Data were represented as the mean ± standard deviation. *p*-value <0.05 was considered statistically significant. The bold values represent the statistically significant values.

**FIGURE 2:**
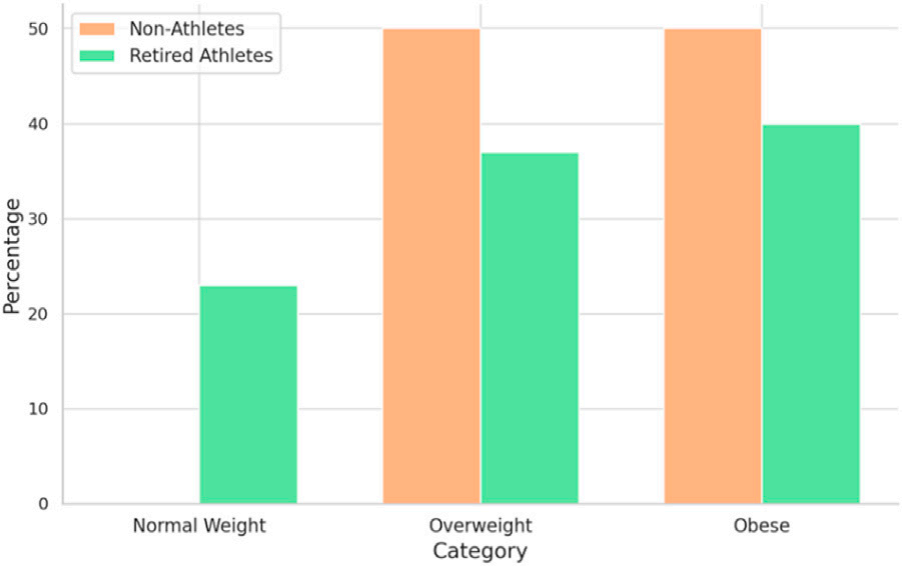
Prevalence of obesity according to the BMI among samples.

The mean BMI of non-athletes was without the normal weight category (30 ± 3.4 kg/m^2^), and the mean BMI of retired athletes was also without the normal weight category (28.6 ± 4.2 kg/m^2^). However, as shown in Figure 2, 50% of non-athletes were overweight, and 50% were obese. For retired athletes, 23% had normal body weight, 37% were overweight, and 40% were obese.

The mean of the waist–hip ratio of non-athletes was 1.35 ± 0.11, and the mean of the waist–hip ratio of retired athletes was 1.27 ± 0.25. According to the waist–hip ratio, 100% of non-athletes and 73.3% of retired athletes have central obesity (waist/hip>1).

### 3.3 Comparison of bone density among retired athletes and non-athletes

**FIGURE 3:**
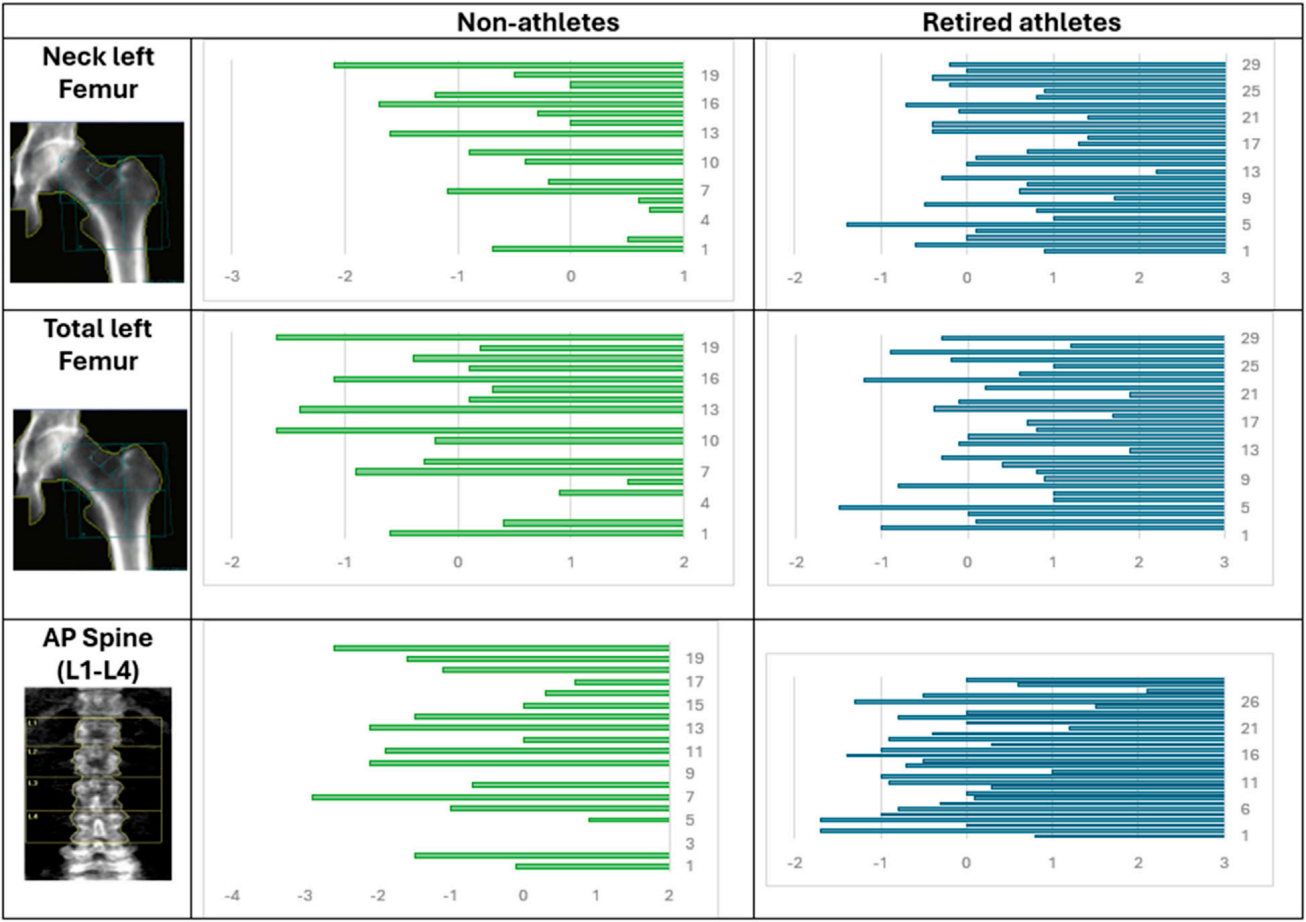
Distribution of the Z-score of the left femur (neck and total) and the AP spine among retired athletes and non-athletes.

Male athletes showed significantly higher Z-scores of the left femur (neck and total) and AP spine with *p*-values of 0.001, 0.047, and 0.0048, respectively, as shown in Table 3. The mean neck Z-score of non-athletes was −0.56 ± 0.84, the total Z-score was 0.279 ± 0.896, and the calculated AP spine Z-score was −1.15 ± 1.17. The mean neck Z-score of retired athletes was 0.32 ± 0.83, the total Z-score was 0.28 ± 0.89, and the calculated AP spine Z-score was −0.15 ± 1.03. Figure 3 shows the distribution of the Z-score of the left femur (neck and total) and AP spine among retired athletes and non-athletes.

**TABLE 3:** Comparison of bone density among samples.

Variable	Non-athletes N = 20 ±(SD)	Retired athletes N = 30 ±(SD)	*p*-value
Neck Z-score (left femur)	−0.56 ± 0.84	0.32 ± 0.83	0.00
Total Z-score (left femur)	−0.29 ± 0.89	0.28 ± 0.89	0.04
Neck T-score (left-femur)	−1.22 ± 0.48	0 ± 0	0.11
Total T-score (left femur)	−1.05 ± 0.82	−0.6 ± 0	0.66
Calculated AP spine Z-score	−1.15 ± 1.17	−0.15 ± 1.03	0.00
Calculated AP spine T-score	−1.08 ± 0.63	0.4 ± 0	0.13
Bone mineral content	2.99 ± 0.36	3.19 ± 0.42	0.08

*Data were represented as the mean ± standard deviation. *p*-value <0.05 was considered statistically significant.

In terms of the neck Z-score, the CI is (−1.38, −0.38), indicating that the observed difference is dependable. The effect size was −1.05, suggesting a large effect. For the total Z-score, the CI is (−0.539, 0.537), which means that the difference might be very small and not practically relevant. The effect size was −0.001, suggesting a small effect size. In terms of the AP spine, the CI is (−1.67, −0.33), indicating that the observed difference is dependable. The effect size was −0.92, suggesting a large effect.

**FIGURE 4:**
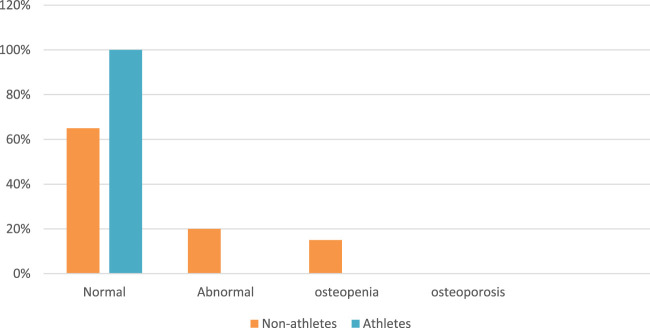
Abnormal bone density, osteopenia, and osteoporosis prevalence among samples.

According to Z-scores and T-scores, 65% of non-athletes have normal bone density, 20% have abnormal bone density, and 15% have osteopenia. For retired athletes, 100% have normal bone density, as shown in Figure 4.

### 3.4 Comparison of lean mass and fat mass among retired athlete and non-athlete male participants

Regarding body composition, as shown in Table 4, there is no significant difference in lean mass among retired and non-athletes (*p*-value > 0.05). Still, retired athletes showed a significantly higher mass in lean legs with *p*-value = 0.03, the mean of lean legs for retired athletes was 22 ± 3.65 kg, and the mean of lean legs for non-athletes was 19.92 ± 2.46 kg. The CI is (0.27, 3.89), indicating that the observed difference is reliable. The effect size was 0.64, suggesting a medium effect.

**TABLE 4:** Comparison of lean mass and fat mass among samples.

Variable	Non-athletes N = 20 ±(SD)	Retired athletes N = 30 ±(SD)	*p*-value
Lean mass (Kg)	57.22 ± 5.68	60.85 ± 8.08	0.09
Arm lean (Kg)	7.67 ± 0.89	8.28 ± 1.31	0.08
Leg lean (Kg)	19.92 ± 2.46	22 ± 3.65	**0.03**
Trunk lean (Kg)	26.03 ± 2.59	27.42 ± 3.35	0.12
Fat mass (Kg)	30.04 ± 7.49	24.11 ± 8.38	**0.01**
Leg fat (Kg)	8.07 ± 1.93	7.03 ± 2.38	0.11
Arm Fat (Kg)	2.94 ± 0.88	2.25 ± 0.75	**0.00**
Trunk Fat (Kg)	17.98 ± 4.82	13.82 ± 5.57	**0.01**

*Data were represented as the mean ± standard deviation. *p*-value <0.05 was considered statistically significant. The bold values represent the statistically significant values.

Non-athletes showed a significantly higher mass in fat mass, arms fats, and trunk fats, with p-values of 0.01, 0.00, and 0.01, respectively. The mean fat mass for non-athletes was 30.04 ± 7.49 kg, and the mean fat mass for retired athletes was 24.11 ± 8.38 kg. The mean arm fat for non-athletes was 2.938 ± 0.884 kg, and the mean arm fat for retired athletes was 2.25 ± 0.75 kg. The mean trunk fat for non-athletes was 17.98 ± 4.82 kg, and the mean trunk fat for retired athletes was 13.82 ± 5.57 kg.

Regarding fat mass, the CI is (1.19, 10.67), indicating that the observed difference is precise and reliable. The effect size was 0.74, suggesting a large effect. For arm fat, the CI is (0.18, 1.19), indicating that the observed difference is precise and reliable. The effect size was 0.86, suggesting a large effect. For trunk fat, the CI is (1.07, 7.25), indicating that the observed difference is precise and reliable. The effect size was 0.79, suggesting a large effect.

### 3.5 Comparison of energy-nutrient intake among retired athlete and non-athlete male participants

Retired athlete male participants showed a significantly higher intake of calories, protein, and carbohydrates with *p*-values of 0.01, 0.00, and 0.04, respectively. The mean daily intake for retired athletes was 1955.16 Kcal/day ±416.27 calories, 98.58 g/day ± 22.01 of protein, and 214.15 g/day ± 76.60 of carbohydrates; the mean daily intake for non-athletes was 1675.25 Kcal/day ±306.64 calories, 80.99 g/day ± 16.85 of protein, and 175.46 g/day ±36.61 of carbohydrates.

The CI for calories (0.05, 0.35) indicates that the true difference in the calorie intake between retired athletes and non-athletes is likely to be between 0.05 and 0.35, with a high level of confidence. The effect size was 0.75, suggesting a large effect. The CI for protein (1.14, 3.27) indicates that the true difference in protein intake could be between 1.14 and 3.27 g, which reinforces the significant difference. The effect size was 0.80, suggesting a large effect. Carbohydrates (18.91, 69.26) reflect greater variability in carbohydrate intake differences. The effect size was 0.49, suggesting a medium effect.

Trans-fat non-athlete male participants showed a significantly higher intake with a *p*-value of 0.00, the mean intake for non-athletes was 0.38 g/day ± 0.65, and the mean for retired athletes was 0.028 g/day ± 0.12. The trans-fat CI (0.09, 0.64) indicates that the actual difference in trans-fat intake is likely between 0.09 and 0.64 g, highlighting a significant difference. The effect size (Cohen’s d) was 1.26, suggesting a very large effect.

In terms of micronutrient intake for vitamins, athletes showed a significantly higher intake of vitamin D, vitamin B1, and biotin, with *p*-values of 0.01, 0.01, and 0.01, respectively. The CIs were (0.08, 0.99); (0.08, 0.28); and (0.62, 3.88), respectively, all indicating a dependable difference. The size effect were (0.77), (0.70), and (0.77), respectively, suggesting a large effect. The mean daily intake for retired athletes was 0.63 mcg/day ±0.31 of vitamin D, 0.87 mg/day ± 0.30 of vitamin B1, and 7.28 mcg/day ± 3.42 of biotin, and the mean of daily intake of non-athletes was 0.39 mcg/day ± 0.28 of vitamin D, 0.67 mg/day ± 0.21 of vitamin B1, and 5.03 mcg/day ± 2.12 of biotin.

However, the non-athlete male participants showed a significantly higher intake of vitamin C, with a *p*-value of 0.02 and a CI of (3.20, 32.72), indicating a reliable difference and an effect size of 0.71, which suggests a large effect. The mean daily intake for non-athletes was 40.21 mg/day of vitamin C ± 22.53, and the mean daily intake for retired athletes was 22.25 mg/day ± 27.04 of vitamin C.

For minerals, retired athletes showed a significantly higher intake of calcium (*p*-value = 0.02, CI= (127.26, 285.64), and effect size = 1.39), chromium (*p*-value = 0.03, CI= (0.12, 1.73), and effect size = 0.74)), iodine (p-value = 0.01, CI=(0.63, 2.36), and effect size = 0.78), iron (*p*-value = 0.007, CI= (2.26, 11.04), and effect size = 0.93), molybdenum (*p*-value = 0.03 < 0.05, CI= (0.24, 2.56), and effect size = 0.62), phosphorus (*p*-value = 0.03, CI= (0.22, 1.86), and effect size = 0.54), and zinc (*p*-value = 0.03, CI= (0.14, 2.57), and effect size = 0.57). All CI values indicate reliable differences, and the effect size ranges from medium to large effects.

The mean daily calcium intake for non-athletes was 336.93 mg ± 81.74, and for retired athletes, it was 445.01 mg ± 182.03. The mean daily chromium intake for non-athletes was 2.77 mcg ± 1.19, and for retired athletes, it was 3.91 mcg ±2.14. The mean daily intakes of iodine, iron, and molybdenum for non-athletes were 37.85 mcg ± 22.99, 10.73 mg ± 3.16, and 6.31 mcg ± 3.44, respectively, and for retired athletes, they were 65.57 mcg ± 39.01, 13.74 mg ± 4.04, and 14.99 mcg ± 17.55, respectively.

As for phosphorus and zinc, the mean daily intakes for non-athletes were 581.329 mg ± 202.17 and 7.22 mg ± 3.19; for retired athletes, they were 712.820 mg ± 199.91 and 9.385 mg ± 3.48. A summary of these findings is shown in Table 5.

**TABLE 5:** Estimated mean daily intakes of energy, macronutrients, and micronutrients using 3-day food records, and the percentage of daily intake compared with the DRI and DGR among the sample.

Energy and micronutrient	Non-athletes N = 20 ±(SD)	Percentage of intake for non-athletes compared with DRI	Retired athletes N = 30 ±(SD)	Percentage of intake for retired athletes compared with DRI	*p*-value
Calories (kcal)	1675.26 ± 306.65	76.1%	1955.16 ± 416.28	88.8%	**>0.01**
Fat calories (kcal)	640.68 ± 147.90	38.2%	700.34 ± 220.99	35.8%	0.29
Saturated fat calories (kcal)	181.29 ± 48.21	10.8%	192.81 ± 81.31	9.9%	0.57
Protein (g)	80.99 ± 16.86	19.3%	98.59 ± 22.01	20.2%	**>0.00**
Carbohydrate (g)	175.46 ± 36.61	41.9%	214.151 ± 76.60	43.8%	**>0.04**
Fiber (g)	11.66 ± 4.39	37.6%	15.023 ± 12.22	48.4%	0.22
Fat (g)	71.54 ± 16.49	92.2%	78.12 ± 24.64	100%	0.30
Saturated fat (g)	20.14 ± 5.36	91.5%	21.42 ± 9.03	97.2%	0.57
Monounsaturated fat (g)	19.49 ± 5.90	31.9%	20.47 ± 8.55	33.5%	0.66
Polyunsaturated fat (g)	11.57 ± 4.81	30%	9.19 ± 5.53	23.8%	0.12
Trans fat (g)	0.38 ± 0.65	38%	0.03 ± 0.12	**3%**	**>0.00**
Cholesterol (mg)	274.42 ± 106.79	91.4%	287.771 ± 104.39	95.9%	0.66
Omega-3 (g)	0.59 ± 0.24	35%	0.59 ± 0.27	34.7%	0.95
Omega-6 (g)	6.47 ± 2.74	404%	5.46 ± 3.25	341%	0.26
Fat-soluble vitamins					
Vitamin A-RAE (RAE)	418.77 ± 566.17	46.5%	270.25 ± 277.69	30%	0.22
Vitamin E-alpha tocopherol (mg)	1.52 ± 1.11	10%	1.74 ± 2.21	11.6%	0.68
Vitamin K (mcg)	47.26 ± 131.59	39.3%	18.51 ± 15.92	15.4%	0.24
Vitamin D-mcg (mcg)	0.39 ± 0.28	0.06%	0.63 ± 0.31	0.10%	**>0.01**
Water-soluble vitamins					
Vitamin B1 (mg)	0.67 ± 0.21	56.2%	0.87 ± 0.30	72.9%	**>0.01**
Vitamin B2 (mg)	0.74 ± 0.44	56.6%	0.86 ± 0.27	66.2%	0.22
Vitamin B3 (mg)	15.58 ± 6.30	-	17.69 ± 7.17	-	0.29
Vitamin B3-NE (mg)	24.68 ± 9.94	154.2%	27.92 ± 10.49	174.5%	0.28
Vitamin B6 (mg)	0.90 ± 0.32	69.3%	1.15 ± 0.66	88.3%	0.12
Vitamin B12 (mcg)	3.26 ± 6.18	139.9%	4.23 ± 4.91	176.2%	0.54
Biotin (mcg)	5.03 ± 2.12	16.7%	7.28 ± 3.42	24.2%	**>0.01**
Vitamin C (mg)	40.21 ± 22.53	44.6%	22.25 ± 27.04	24.7%	**>0.02**
Folate, DFE (mcg)	297.24 ± 140.75	74.3%	362.36 ± 138.62	90.5%	0.11
Pantothenic acid (mg)	3.29 ± 1.58	65.7%	3.466± 1.18	69.3%	0.65
Minerals					
Calcium (mg)	336.93 ± 81.74	33.69%	445.01 ± 182.03	44.5%	**>0.02**
Chromium (mcg)	2.77 ± 1.19	7.9%	3.91 ± 2.14	11.1%	**>0.03**
Copper (mg)	0.69 ± 1.00	77.2%	0.66 ± 0.83	73.2%	0.89
Iodine (mcg)	37.85 ± 22.99	25.2%	65.57 ± 39.01	43.7%	**>0.01**
Iron (mg)	10.73 ± 3.16	134.1%	13.74 ± 4.04	171.7%	**>0.01**
Magnesium (mg)	118.71 ± 48.90	28.2%	136.08 ± 48.70	32.4%	0.22
Manganese (mg)	1.47 ± 0.56	63.7%	1.82 ± 1.16	79.1%	0.12
Molybdenum (mcg)	6.31 ± 3.44	14.02%	14.99 ± 17.55	33.3%	**>0.03**
Phosphorus (mg)	581.33 ± 202.17	83.04%	712.82 ± 199.91	101.8%	**>0.03**
Potassium (mg)	1165.06 ± 397.81	34.2%	1278.60 ± 579.05	63.7%	0.45
Selenium (mcg)	57.51 ± 23.95	104.5%	70.74 ± 30.79	128.6%	0.11
Sodium (mg)	2574.17 ± 997.97	111.9%	2614.48 ± 1076.73	113.6%	0.89
Zinc (mg)	7.22 ± 3.19	65.6%	9.38 ± 3.48	85.3%	**>0.03**
Others					
Caffeine (mg)	19.09 ± 30.49	4.7%	9.50 ± 19.75	2.3%	0.18

Abbreviations: RAE, retinol activity equivalent; RAE, 1 mcg retinol, 12 mcg beta-carotene, 24 mcg alpha-carotene, 24 mcg beta-cryptoxanthin; NE, niacin equivalent; NE, 1 mg of niacin = 60 mg of tryptophan; DFE, dietary folate equivalent; DFE, 1 mcg food folate, 0.6 mcg food fortified with folic acid. *Data were represented as the mean ± standard deviation. *p*-value <0.05 was considered statistically significant. The bold values represent the statistically significant values.

### 3.6 Assessment of energy-nutrient intake among retired athlete and non-athlete male participants

The percentage of daily intake of macronutrients was calculated by multiplying the grams of each macronutrient by the calorie per gram for that nutrient; each gram of protein and CHO contributes 4 calories to the caloric total, and each gram of fat contributes 9 calories, divided by the mean energy intake of male athletes and non-athletes . By comparing the percentage of protein intake per day from the total energy intake, which was 20.2% in retired athletes vs 19.3% in non-athletes, the study found that all participants were in the normal range of Acceptable Macronutrient Distribution Range (AMDR), which is 10%–35%. The percentage of carbohydrate intake per day among retired athletes was 43.8%, and among non-athletes, it was 41.9%, which was within the AMDR, 45%–65%.

**FIGURE 5:**
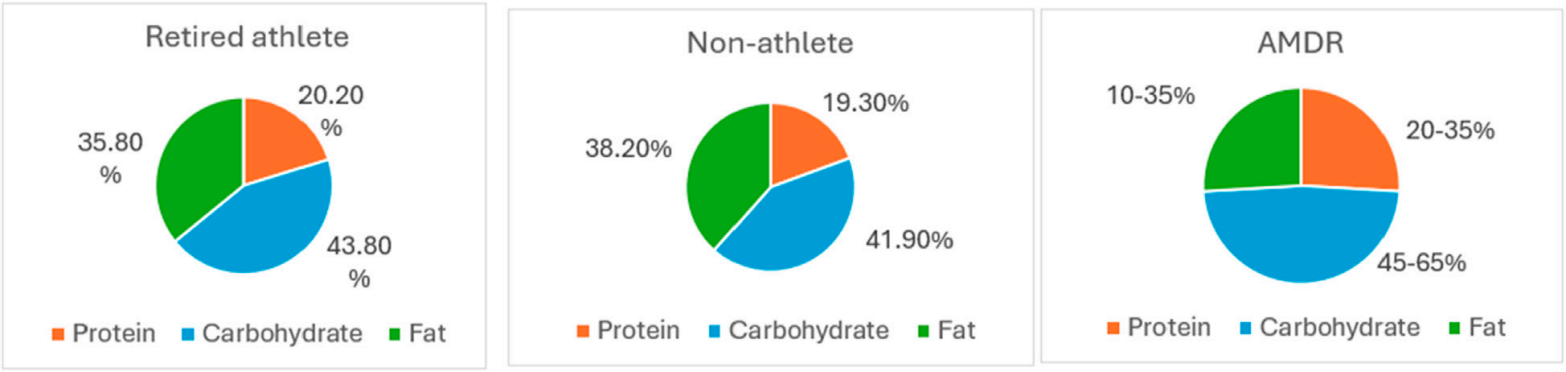
Percentage of protein, carbohydrate, and fat intake compared with the AMRD.

The percentages of daily fat intake for retired athletes and non-athletes were 35.8% and 38.2%, respectively, of the total energy intake. For retired athletes, this figure is at the upper limit, while for non-athletes, it indicates a higher fat intake compared to the AMDR, 20%–35%. In addition, the saturated fat intake within the DGR for retired and non-athletes was 10.8% and 9.9%, respectively. Figure 5 shows the percentage of protein, carbohydrate, and fat intake compared with the AMDR.

For dietary fiber, the mean daily intake was 11.659 g/day ±4.39 for non-athletes and 15.023 g/day ±12.22 for retired athletes; both groups did not meet the adequate intake (AI) for fiber, which is equal to 31 g/day. For omega-3 and omega-6, the non-athletes and retried athletes met only 35% and 34.7% of the AI for omega-3 fatty acid, respectively, while for omega 6, both groups exceeded the AI by 404% and 341%, respectively.

Regarding micronutrients, fat-soluble vitamins (A, D, E, and K) were below the recommended level for both groups; the mean daily intake of vitamins E and D was much lower than the RDA. The water-soluble vitamins (B1, B2, B6, folate, B12, and C) were below the RDA, except for vitamin B3 and B12, which exceeded the recommended level among both groups; the mean intakes of vitamin B3 for non-athletes and retired athletes were 154.2% and 174.5% of RDA. The mean intakes of vitamin B12 for non-athletes and retired athletes were 139.9% and 176.2% of the RDA. As for minerals, calcium, chromium, copper, iodine, magnesium, manganese, molybdenum, phosphorus, potassium, and zinc were below the RDA in both groups. Otherwise, iron, sodium, and selenium exceeded the RDA among both groups. The mean daily intake of chromium was much lower than AI as chromium daily intake met the (7.9% non-athletes and 11.1% retired athletes) requirement of AI. A summary of these findings is shown in Table 5.

### 3.7 Comparison of Perceived Stress Scale (PSS-14), WHOQOOL (GHQ-26), Subjective Happiness Scale, and Insomnia Severity Index (ISI-5) among retired athlete and non-athlete male participants

There were statistically significant mean differences between retired and non-athletes of perceived stress levels with a CI of (0.052, 0.368), which indicates that the difference was dependable, and an effect size of 0.70 suggests a large effect. In contrast, there were no statistically significant mean differences between the groups in quality of life, subjective happiness, and the Insomnia Severity Index (*p*-value >0.05), as shown in Table 6. The mean perceived stress level for non-athletes and retired athletes was 2.15 (SD = 0.29) and 2.36 (SD = 0.32) with a *p*-value of 0.03.

**TABLE 6:** Mean of quality of life, perceived stress, subjective happiness scores, the Insomnia Severity index, and physical activity among the sample.

Variable	Non-athletes	Retired athletes	*p*-value
Quality of life	3.43 ± 0.53	3.67 ± 0.42	0.09
Perceived stress	2.15 ± 0.29	2.36 ± 0.32	**>0.03**
Subjective happiness	4.02 ± 0.84	3.59 ± 0.86	0.09
Insomnia Severity Index	1.22 ± 0.62	1.17 ± 0.62	0.80
Days of high-intensity exercise	0 ± 0	1.53 ± 1.61	**>0.00**
Duration of high-intensity exercise (min)	0 ± 0	31.33 ± 34.06	**>0.00**
Days of moderate-intensity exercise	0.1 ± 0.45	1.80 ± 1.88	**>0.00**
Long moderate-intensity exercise (min)	1 ± 4.47	31.83 ± 32.99	**>0.00**
Days of walking	3.5 ± 2.68	4.37 ± 1.77	0.17
Duration of walking (min)	26.11 ± 26.54	35.83 ± 30.34	0.25
Duration of sitting (hr.)	2.85 ± 2.83	2.97 ± 1.87	0.86

*Data were represented as the mean ± standard deviation. *p*-value <0.05 was considered statistically significant. General health and subjective happiness: the higher the score, the better the parameter. Perceived stress and the Insomnia Severity Index: The higher the score, the worse the parameter. The bold values represent the statistically significant values.

The mean quality of life scores for non-athletes and retired athlete male participants were 3.43 (SD = 0.53) and 3.67 (SD = 0.42), respectively. The mean subjective happiness score was 4.02 (SD = 0.84) for non-athletes vs. 3.59 (SD = 0.86) for retired athletes. In addition, the mean insomnia severity score was 1.22 (SD = 0.62) for non-athletes vs. 1.17 (SD = 0.62) for retired athletes.

By evaluating the quality of life (QOL) scores for both retired athletes and non-athletes, it is evident that both groups demonstrate a good level of quality of life. Regarding insomnia, 30% of non-athletes exhibited a low level of insomnia, while 25% experienced a moderate level of insomnia. For retired athletes, 30% reported a low level of insomnia, and 10% indicated a moderate level of insomnia.

Among non-athletes, 65% experienced a moderate level of stress, while 20% reported a high level of stress. Among retired athletes, 90% reported a moderate stress level, and 10% experienced a high stress level. When assessing subjective happiness, the mean score for non-athletes exceeded 4, indicating that they are generally happy. The proportion of male participants who described themselves as happy was 23% for retired athletes, compared to 45% for non-athletes.

### 3.8 Comparison of physical activity among non-athlete and retired athlete male participants

In terms of high- and moderate-intensity exercise, retired athletes showed significantly more days and longer durations of both types of exercise. The mean days and length of high-intensity exercise for retired athletes were 1.53 days (SD = 1.61) and 31.33 min (SD = 34.06), respectively.

The mean days and length of moderate-intensity exercise for retired athletes were 1.80 days, (SD = 1.88) and 31.83 min, (SD = 32.99), and for non-athletes, they were 0.1 days (SD = 0.45) and 1 min, (SD = 4.47), respectively. In contrast, there were no significant differences between non-athletes and retired athletes in terms of days and durations of walking and sitting. A summary of these findings is shown in Table 6.

Overall, 100% of non-athletes had insufficient physical activity according to a guideline of IPAQ. As for retired athletes, 30% had insufficient physical activity, 50% had minimum physical activity, and 20% had high physical activity.

### 3.9 Detected correlations between variables

#### 3.9.1 Detected correlations between current weight and other variables

Supplementary Table S1 illustrates the correlation between current weight and other variables. The current weight was positively correlated with bone mineral content (BMC) (r = 0.59 and *p*-value = 0.01) for non-athletes.

For retired athletes, the current weight showed a significant positive correlation and difference with weight in the last competition (r = 0.59 and *p*-value = 0.01), daily intake of calories (r = 0.59 and *p*-value = 0.01), daily intake of carbohydrates (r = 0.59 and *p*-value = 0.01), molybdenum intake (r = 0.43* and *p*-value = 0.02), sodium intake (r = 0.47** and *p*-value = 0.01), and BMC (r = 0.65** and *p*-value = 0.00) and negative correlation with pantothenic acid (r = −0.36 and *p*-value = 0.04) and high-intensity activity (r = −0.38* and *p*-value = 0.04).

#### 3.9.2 Detected correlations between bone density variables and other variables

Supplementary Table S2 shows that the neck Z-score (left femur) showed a negative correlation with omega-3 intake (r = −0.55* and *p*-value = 0.03) and omega-6 intake (r = −0.56* and *p*-value = 0.02) for non-athletes and a negative correlation with omega-6 intake (r = −0.39** and p-value = 0.03) for retired athletes. In addition, the neck Z-score showed a positive correlation with the perceived stress level (r = 0.44* and p-value = 0.02) for athletes. Total Z-score (left femur) showed a negative correlation with monounsaturated fat (r = −0.54* and *p*-value = 0.03), polyunsaturated fat intake (r = −0.54* and *p*-value = 0.03) for non-athletes and omega-3 intake (r = −0.62** and *p*-value = 0.01) (r = −0.38* and *p*-value = 0.04), omega-6 intake (r = −0.68** and *p*-value = 0.00) (r = −0.47** and *p*-value = 0.01), and vitamin B3 intake (r = −0.50*and *p*-value = 0.05) (r = −0.41* and *p*-value = 0.03) for non-athletes and retired athletes. Total Z-score showed positive correlation with current weight (r = −0.50* and *p*-value = 0.04) and perceived stress (r = −0.55* and *p*-value = 0.03) for retired athletes. For the neck, the T-score (left femur) showed a positive correlation with molybdenum intake (r = 0.95** and *p*-value = 0.04) and a negative correlation with caffeine intake (r = 0.99** and *p*-value = 0.01) for non-athletes. Total T-score (left femur) showed a positive correlation with molybdenum (r = 0.95* and *p*-value = 0.04) and walking (r = 0.98* and *p*-value = 0.01) for non-athletes. The correlation coefficient of the T-score (neck and total) for retired athletes could not be measured because one participant was assessed by the T-score in retired athletes.

In terms of the calculated Z-score, the AP spine showed a negative correlation with omega-6 intake (r = −0.49* and *p*-value = 0.01) and vitamin B3 intake (r = −0.383* and *p*-value = 0.04). It showed a positive correlation with stress (r = 0.489* and *p*-value = 0.01) for retired athletes. In terms of the calculated T-score, the AP spine showed a positive correlation with weight before 10 years (r = 0.91* and *p*-value = 0.03) and showed a negative correlation with caffeine (r = 0.96* and *p*-value = 0.01) for non-athletes.

BMC showed a positive correlation with the current weight (r = 0.59* and *p*-value = 0.01) and lean mass (r = 0.73* and *p*-value = 0.00) for non-athletes. Retired athletes showed a positive correlation with the current weight (r = 0.65** and *p*-value = 0.00), weight in the last competition (r = 0.66* and *p*-value = 0.00), fat mass (r = 0.39* and *p*-value = 0.03), lean mass (r = 0.73** and *p*-value = 0.00), manganese intake (r = 0.39* and *p*-value = 0.03), and BMI (r = 0.42* and *p*-value = 0.02). They were negatively correlated with omega-6 (r = −0.49** and *p*-value = 0.01).

#### 3.9.3 Detection of the correlation between the intensity of physical activity, fat mass, and lean mass

Table 7 shows that there is no significant correlation between physical activity and fat mass and lean mass for both groups, with a *p*-value >0.05.

**TABLE 7:** Correlation between physical activity, fat mass, and lean mass among samples.

Group	Variable		High-intensity activity (min)	Moderate-intensity activity (min)	Walking time (min)	Sitting time (hr.)
Non-athletes	Fat mass	r	. a	−0.06	0.14	−0.03
		*p*-value	-	0.81	0.58	0.89
	Lean mass	r	. a	0.21	−0.10	−0.25
		*p*-value	-	0.36	0.68	0.29
Retired athletes	Fat mass	r	. a	−0.06	0.14	−0.03
		*p*-value	-	0.81	0.58	0.89
	Lean mass	r	. a	0.21	−0.10	−0.25
		*p*-value	-	0.36	0.68	T10.29

Abbreviations: Pearson’s correlation; r. *p*-value <0.05 was considered statistically significant*. Correlation is significant at the 0.05 level, **. The correlation is significant at the 0.01 level. a. Cannot be computed because at least one of the variables is constant.

## 4 Discussion

Our study aimed to assess bone density and weight changes among retired elite male martial arts athletes in Amman, along with body composition, physical activity, quality of life, insomnia severity, subjective happiness, macro- and micronutrient intake, and perceived stress.

### 4.1 Macronutrient and micronutrient intake

Adequate nutrition intake of both macronutrients and micronutrients is essential for maintaining good health throughout life for all populations (Puwanant et al., 2022). This is particularly critical for retired athletes as proper nutrition during retirement supports muscle and bone health and promotes mental wellbeing (Moreno-Agostino et al., 2020; Thomas et al., 2016).

In our study, based on the analysis of a 3-day food record, retired athlete male participants showed a significantly higher intake of macronutrients, while non-athlete male participants showed a significantly higher intake for trans-fat. In terms of vitamins, retired athletes showed a significantly higher intake of vitamin D, vitamin B1, and biotin, whereas non-athlete male participants showed a higher vitamin C intake. For minerals, retired athletes showed a significantly higher intake of calcium, chromium, iodine, iron, molybdenum, phosphorus, and zinc.

Overall, both retired athletes and non-athletes met their requirements for macronutrients but did not meet their requirements for most micronutrients; only omega-3, omega-6, vitamin B3, and vitamin B12 exceeded the recommendation. This is consistent with the findings of Yao et al. (2020), who showed that 79.13% of 122 former elite athletes did not meet the worldwide nutritional guidelines, and with those of Gouttebarge et al. (2015), who discovered that 42% had adverse nutritional behaviors.

Many athletes follow a strict and structured diet during their careers to enhance performance (Yao et al., 2020; Silva Santos et al., 2016). After retirement, these nutritional needs are modified; some athletes decrease their PA but do not adjust their dietary habits accordingly (Yao et al., 2020). Additionally, the chance of eating unhealthy foods has increased for retired athletes compared to their competitive years due to removing the constraints (Altowerqi et al., 2020). This could be due to several contributing factors, such as psychological influences like emotional eating as a response to the stress of transitioning to post-athletic life or simply a sense of freedom from previously imposed restrictions. Reduced access to professional dietary guidance and changes in energy expenditure may further exacerbate these habits (Hadiyan and Cosh, 2019).

Economic factors also play a significant role in dietary behaviors. During their careers, many athletes have access to financial resources and professional dietary support, which enable them to maintain a balanced diet (Martín-Rodríguez et al., 2024; Schmid et al., 2023) These factors, when combined, help explain our findings.

### 4.2 Physical activity

Regular physical activity has long been recognized as an integral component of a healthy lifestyle, providing multiple health benefits; it helps prevent diseases such as stroke, heart disease, diabetes, and osteoporosis (Adamson et al., 2022; World Health Organization, 2018). According to the WHO, there are specific minimum physical activity recommendations required for various age groups to achieve optimal health (World Health Organization, 2018). Children and adolescents should engage in at least 60 min of moderate-intensity physical activity daily, primarily aerobic exercises, supplemented by at least 30 min of vigorous-intensity physical activity. Adults aged 18–64 need to participate in 150–300 min of moderate-intensity aerobic physical activity; 75–150 min of vigorous-intensity aerobic physical activity ; or an equivalent combination of vigorous- and moderate-intensity activities all over the week (World Health Organization, 2022; Piercy et al., 2018; World Health Organization, 2018)

In our study, retired athletes showed practicing high- and moderate-intensity exercises more than non-athletes. In contrast, there are no significant differences between non-athletes and retired athletes in walking and sitting. Current levels of high-intensity exercise showed a significant correlation with weight in retired athletes.

According to a guideline of IPAQ, 100% of non-athletes had insufficient physical activity. As for retired athletes, 30% had insufficient physical activity, 50% had minimum physical activity, and 20% had high physical activity. This is consistent with what was reported by Naderyan et al. (2019), who found that only 4.9% of former Iranian athletes walk regularly. Furthermore, 42% of former athletes did not engage in moderate-intensity physical activity lasting at least 10 min. Regarding intense activity, 52.9% of the participants had never engaged in vigorous-intensity physical activity lasting at least 10 min, and none reported doing so regularly. However, this is different from what was reported by Plateau et al. (2017), who found that retired athletes had physical activity levels consistent with recommendations.

Two hypotheses may explain the higher physical activity levels of retired athletes. Several studies suggest that many retired elite athletes continue to participate and be involved in sports as coaches, keeping them more active. Additionally, many athletes express that they enjoy exercising for pleasure after retirement, which helps them maintain higher activity levels than others (Plateau et al., 2017).

For the 30% of individuals with insufficient physical activity among retired athletes, this can be attributed to several factors. Chronic injuries sustained during their athletic careers often limit their ability to engage in regular exercise (Hadiyan and Cosh, 2019). Additionally, shifts in life priorities, such as focusing on family or new career paths, can deprioritize physical activity. Psychological challenges, including the loss of identity and purpose after retiring from sports, further impact motivation to stay active. The absence of competitive goals and a supportive team environment also exacerbates this decline (Hadiyan and Cosh, 2019). Moreover, the natural aging process brings physiological changes, such as reduced energy levels, decreased endurance, and slower recovery, making it more difficult to maintain high levels of physical activity (Burtscher et al., 2022).

### 4.3 General quality of health, insomnia severity, subjective happiness, and perceived stress

In our study, retired athletes have higher perceived stress than non-athletes. In contrast, there were no statistically significant mean differences between the groups in general health, subjective happiness, and the Insomnia Severity Index. This is consistent with what was reported by Jónsdóttir et al. (2022), who found that quality of life was not significantly different between athlete and non-athlete female participants.

Retirement from sports can be a significant source of stress for athletes, particularly those who are unplanned and unprepared for life after sports and do not have any alternative career (Knights et al., 2018; Hatamleh and Mazin, 2013). Uncertainty about the future, financial concerns, and the challenge of adjusting to a new routine can all contribute to increased stress during this period (Hatamleh and Mazin, 2013).

The quality of an athlete’s retirement experience plays a crucial role in influencing their perceived stress levels (Knights et al., 2018; Hatamleh and Mazin, 2013). Social and organizational support are essential during the transition phase; athletes who feel unsupported or isolated during this time are more likely to experience an increased level of perceived stress (Knights et al., 2018)

Overall, both non-athletes and retired athletes have a good quality of life, with a mean score of 3.43 ± 0.53 out of 5 for non-athletes and 3.67 ± 0.42 out of 5 for retired athletes. For subjective happiness, the mean score for non-athletes exceeded 4, indicating that they are generally happy. The proportion of male participants who described themselves as happy was 23% for retired athletes, in contrast to 45% for non-athletes, and this finding differs from what was found by Lewandowska et al. (2017), who reported that 56.1% of retired athletes described themselves as happy.

For perceived stress among non-athletes, 65% experienced moderate stress, while 20% reported high levels of stress. Among retired athletes, 90% reported a moderate stress level, and 10% experienced a high-stress level, and this is consistent with Gouttebarge et al. (2015), who reported that 16% of former footballers had perceived stress.

Regarding insomnia, 30% of non-athletes exhibited a low level of insomnia, while 25% of non-athletes experienced moderate insomnia. Of retired athletes, 30% reported a low level of insomnia, and 10% indicated a moderate level of insomnia. This is consistent with what was found by Gouttebarge et al. (2019), who reported that 20.9% of retired athletes reported sleep disturbances.

### 4.4 Weight, BMI, waist-hip ratio, and body composition

In our study, there is no significant difference in the current weight between non-athletes and retired athletes. Still, there is a significant difference between the weight before 10 years for non-athletes and those in the last competition for retired athletes.

The current weight of retired athletes positively correlates with their weight in the last competition, indicating that retired athletes experience greater weight changes than non-athletes. The mean weight gain among retired athletes was 18.55 kg, and the mean weight gain among non-athletes was 4.3 kg. In addition, these changes also have a positive correlation with calorie intake and carbohydrate intake. This contradicts the observations of Marquet et al. (2013), who found that it does not induce massive weight gain after retirement, while this is consistent with what was reported by Emami et al. (2018), who found that retired Iranian male athletes had significantly higher weight and body mass index, with an average increase of 12 kg compared to non-athletes.

There is no significant difference in the BMI and waist/hip ratio. However, 50% of non-athletes were overweight, and 50% were obese. Of retired athletes, 23% had a normal body weight, 37% were overweight, and 40% were obese, and this contradicts the findings reported by Marquet et al. (2013), who found that former French elite athletes in weight category sports had a lower BMI than the general population in the same age.

Athletes engage in high levels of physical activity, which necessitates an increased calorie intake. After retirement, however, many of them reduce their physical activity, which leads to decreased energy expenditure (Flack et al., 2023). The mismatch between energy intake and expenditure is one of the factors contributing to weight gain after retirement (Yao et al., 2020) Additionally, aging naturally reduces resting metabolic rates (RMRs) and leads to hormonal changes, such as decreased levels of testosterone and growth hormone, which further exacerbate the risk of weight gain by promoting fat storage and reducing muscle mass (Zampino et al., 2020).

Psychological challenges also play a significant role in weight gain among retired athletes. Many athletes turn to emotional eating as a coping mechanism, which further elevates the risk of weight gain (Zorzi and Zandonai, 2024). Given the multifactorial nature of weight changes in retired athletes, a balanced approach to physical activity and nutrition is essential. Seeking professional guidance from healthcare providers, dietitians, and sports scientists can help retired athletes navigate this transition and maintain a healthy weight (Thomas et al., 2016)

Regarding body composition, there is no significant difference in lean mass among retired and non-athletes, but retired athletes showed a significantly higher mass in lean legs. Non-athletes showed a significantly higher mass in fat mass, arm fats, and trunk fats.

### 4.5 Bone density

This study demonstrated that bone mineral density (BMD) and bone mineral content levels were significantly higher in martial arts retired elite athletes in different body regions than in non-athlete male individuals of the same age. This is consistent with what was reported by Hagman et al. (2018), who found higher bone mineral density in former elite athletes than in non-athletes.

The bone density variables and BMC showed a negative correlation with omega-3 and omega-6, and this result contradicts the findings reported by Donlon et al. (2018), who used omega-3 in the prevention process of osteoporosis, and Casado-Díaz et al. (2013), who found that omega-3 and omega-6 protect against bone loss. The bone density variables negatively correlated with monounsaturated and polyunsaturated fatty acids. This result contradicts the observations of Fang et al. (2023), who found a positive correlation between monounsaturated and polyunsaturated fatty acids and bone density. The negative correlation of bone density variables with vitamin B3 (niacin) is consistent with what was reported by Carbone et al., 2019, who found that niacin has a negative association with hip BMD.

The bone density variables showed a positive correlation with perceived stress; this result contradicts the findings of Pedersen et al. (2016), who reported a negative correlation between bone density and perceived stress.

BMD is derived by dividing BMC by the bone area (Tian et al., 2016). BMD did not show a significant correlation with any micronutrient that may affect bone density, such as calcium or vitamin D, but showed a significant correlation with the current weight or weight in the last competition. BMC has shown a positive correlation with weight, lean, and fat mass. This is consistent with what was reported by Evans et al. (2015), who found that obese people had higher bone density at all measured sites, and this can justify the findings reported by Lecka-Czernik et al. (2015), who found that a high bone mass in obesity results from the positive effect on bone formation by mechanical load. This is also consistent with what was reported by Jeddi et al. (2015), who found a positive correlation between lean mass and fat mass and BMD, and they justify it by mechanical loads and hormonal effects, such as the increased conversion of androstenedione to estrogen and the high circulating level of leptin.

Therefore, high bone mineral density observed among retired athletes in this study was assessed with the hypothesis that mechanical forces from physical activity before retirement, along with weight and lean mass, contributed to this outcome. Because current physical activity for retired athletes did not show any significant correlation with BMD and BMC and fat mass was higher in non-athletes, it did not explain high BMD and BMC in our study.

Bone is susceptible to mechanical forces from physical activity and weight from both gravitational loading (such as impact) and muscular contraction (Strope et al., 2015). Osteocytes, which are bone cells, experience these mechanical forces as cell deformation, variations in the stress of extracellular fluid, electric fields, and pressure slopes at the cellular level (Hart et al., 2017; Strope et al., 2015). By modulating bone production and resorption, osteocytes interact with osteoclasts and osteoblasts to alter bone geometry and material qualities and become stronger or weaker (Strope et al., 2015).

In terms of muscles, bones and muscles are mechanically, biochemically, and molecularly linked. The strength of bone and muscle mass are proportional (Santos et al., 2017). Muscles connect to bones via tendons, creating a system of levers that facilitate movement. They exert contractile forces on the skeleton, providing the primary voluntary stimulus for bone movement, which is stronger than the effects of gravity (Hart et al., 2017). Additionally, muscles and bones communicate through endocrine and paracrine signaling, where secreted factors influence each other, nearby tissues, and distant organs (Hart et al., 2017).

### 4.6 Conclusion

In conclusion, this study showed that retired athletes had higher bone density than non-athletes; all retired athletes have normal bone density and higher BMC compared to non-athletes. In terms of weight changes, retired athletes show a significantly higher difference in weight, indicating that they gain more weight than non-athletes, but there is no significant difference in the current weight between the two groups, both of which are classified as overweight and obese. In terms of dietary intake, retired athletes and non-athletes meet their requirements of macronutrients but do not meet their requirements for most micronutrients; in addition, retired male athlete participants showed a significantly higher intake of macronutrients. In terms of physical activity, all non-athlete participants had insufficient physical activity according to the guideline of IPAQ. As for retired athletes, 30% had insufficient physical activity, 50% had minimum physical activity, and 20% had high physical activity. In terms of quality of life, subjective happiness, insomnia, and perceived stress, both have a good quality of life and happiness, and both groups have low levels of insomnia and perceived stress. However, retired athletes had significantly higher levels of perceived stress.

Based on these findings, the following recommendations are proposed:

1. Raise awareness: Implement educational programs to increase awareness of the health benefits of physical activity among non-athletes and young individuals in the Middle East.
2. Enhance dietary practices: In general, recommend dietary improvements for all populations, especially in the Middle East, to enhance their nutritional status, which is essential for maintaining healthy body weight and bone health. Regarding retired athletes, develop nutrition plans to support effective weight management and sustain the benefits of pre-retirement physical activity on bone health.
3. Increase physical activity among the population and promote ongoing physical activity: Advocate for initiatives that promote regular physical activity among the general population to improve their overall health, particularly in terms of weight management and bone strength. In terms of retired athletes, encourage them to continue engaging in physical activity as a critical factor in preserving bone health and managing body weight.
4. Expand research on martial arts and sports: Encourage more research focused on the impact of martial arts and other sports on weight changes and bone health among retired athletes, particularly in the Middle East, to emphasize sports’ effects and mechanisms.
5. Investigate gender-specific impacts: Focusing on conducting research that examines the effects of martial arts and other sports on weight and bone health among retired female athletes to address potential gender-specific differences and needs.

### 4.7 Limitation of the study

This study has a few limitations: the sample was insufficient to study the effect of each type of MA on bone and weight change; a small sample size may reduce the statistical power of the study, making it challenging to identify significant differences or associations. This limitation can also cause overestimation or underestimation of results, which might not be generalizable to a larger population. Due to the budget, the participants were asked if they have vitamin D deficiency or any problems in thyroid gland instead of conducting blood tests to determine if there was vitamin D deficiency or any problems in thyroid gland. Self-reported data are liable to several biases, including recall bias and social desirability bias, which may lead participants to overstate or understate their health behaviors or conditions. These biases can affect the reliability and validity of the study’s findings. In addition, there are few research studies on the impact of sports and MA, especially on weight, bone density, and quality of life.

## Data Availability

The raw data supporting the conclusions of this article will be made available by the authors, without undue reservation.
