# Supplementary material for: Comprehensive health assessment of retired martial arts athletes: bone density, dietary intake, physical activity, and wellbeing
Source: Front Aging. 2025 Feb 3;6:1513936. doi: 10.3389/fragi.2025.1513936 (PMC11830723; doi:10.3389/fragi.2025.1513936)
Supplement: Supplementary file 1 [file Table1.docx]

Supplementary Material

## Supplementary Tables

**Supplementary Table 1.** Correlation between current weight with age, weight before 10 years/weight in last competition, energy and nutrient intake, questionnaires, and physical activity among a sample.

| **Variables** |  | **Current weight for non-athletes** | **Current weight for retired athletes** |
| --- | --- | --- | --- |
| **Age** | **r** | 0.13 | 0.16 |
|  | **P-value** | 0.57 | 0.38 |
| **Weight before 10 years** | **r** | 0.18 | 0.65** |
|  | **P-value** | 0.43 | **>0.00** |
| **Calories (kcal)** | **r** | 0.09 | 0.37* |
|  | **P-value** | 0.70 | 0.03 |
| **Protein(g)** | **r** | 0.03 | 0.10 |
|  | **P-value** | 0.87 | 0.57 |
| **Carbohydrate (g)** | **r** | 0.15 | 0.49** |
|  | **P-value** | 0.50 | **>0.00** |
| **Fat (g)** | **r** | -0.01 | 0.01 |
|  | **P-value** | 0.98 | 0.93 |
| **Saturated Fat (g)** | **r** | 0.002 | -0.05 |
|  | **P-value** | 0.99 | 0.79 |
| **Monounsaturated Fat (g)** | **r** | -0.19 | -0.24 |
|  | **P-value** | 0.41 | 0.19 |
| **Polyunsaturated Fat (g)** | **r** | -0.27 | -0.34 |
|  | **P-value** | 0.25 | 0.07 |
| **Trans Fat (g)** | **r** | 0.18 | -0.14 |
|  | **P-value** | 0.45 | 0.46 |
| **Cholesterol (mg)** | **r** | -0.08 | -0.25 |
|  | **P-value** | 0.74 | 0.18 |
| **Omega3 (g)** | **r** | -0.32 | -0.12 |
|  | **P-value** | 0.17 | 0.53 |
| **Omega6 (g)** | **r** | -0.29 | -0.22 |
|  | **P-value** | 0.21 | 0.24 |
| **Vitamin A-RAE (RAE)** | **r** | -0.21 | -0.02 |
|  | **P-value** | 0.37 | 0.90 |
| **Vitamin E-alpha Tocopherol (mg)** | **r** | -0.23 | 0.36 |
|  | **P-value** | 0.33 | 0.053 |
| **Vitamin K (mcg)** | **r** | -0.15 | -0.04 |
|  | **P-value** | 0.54 | 0.85 |
| **Vitamin D-mcg (mcg)** | **r** | -0.11 | -0.25 |
|  | **P-value** | 0.65 | 0.18 |
| **Vitamin B1 (mg)** | **r** | -0.19 | 0.30 |
|  | **P-value** | 0.43 | 0.11 |
| **Vitamin B2 (mg)** | **r** | -0.19 | -0.25 |
|  | **P-value** | 0.42 | 0.18 |
| **Vitamin B3-NE (mg)** | **r** | -0.15 | -0.16 |
|  | **P-value** | 0.52 | 0.39 |
| **Vitamin B6 (mg)** | **r** | -0.22 | -0.04 |
|  | **P-value** | 0.34 | 0.84 |
| **Vitamin B12** | **r** | -0.09 | -0.20 |
|  | **P-value** | 0.72 | 0.29 |
| **Biotin (mcg)** | **r** | 0.24 | -0.14 |
|  | **P-value** | 0.31 | 0.46 |
| **Vitamin C (mg)** | **r** | -0.10 | 0.19 |
|  | **P-value** | 0.66 | 0.29 |
| **Folate, DFE (mcg)** | **r** | -0.10 | 0.21 |
|  | **P-value** | 0.67 | 0.27 |
| **Pantothenic acid (mg)** | **r** | -0.22 | -0.36* |
|  | **P-value** | 0.36 | **>0.04** |
| **Calcium (mg)** | **r** | -0.25 | -0.04 |
|  | **P-value** | 0.29 | 0.82 |
| **Chromium (mcg)** | **r** | -0.04 | 0.09 |
|  | **P-value** | 0.87 | 0.62 |
| **Copper (mg)** | **r** | -0.11 | -0.08 |
|  | **P-value** | 0.66 | 0.68 |
| **Iodine (mcg)** | **r** | -0.08 | -0.19 |
|  | **P-value** | 0.73 | 0.31 |
| **Iron (mg)** | **r** | -0.27 | 0.21 |
|  | **P-value** | 0.25 | 0.26 |
| **Magnesium (mg)** | **r** | -0.21 | 0.21 |
|  | **P-value** | 0.38 | 0.26 |
| **Manganese (mg)** | **r** | -0.29 | 0.37 |
|  | **P-value** | 0.22 | 0.05 |
| **Molybdenum (mcg)** | **r** | -0.02 | 0.43* |
|  | **P-value** | 0.93 | **>0.02** |
| **Phosphorus (mg)** | **r** | -0.18 | -0.02 |
|  | **P-value** | 0.45 | 0.92 |
| **Potassium (mg)** | **r** | -0.131 | 0.16 |
|  | **P-value** | 0.58 | 0.40 |
| **Selenium (mcg)** | **r** | -0.13 | -0.17 |
|  | **P-value** | 0.58 | 0.37 |
| **Sodium (mg)** | **r** | 0.04 | 0.47** |
|  | **P-value** | 0.86 | **>0.01** |
| **Zinc (mg)** | **r** | -0.12 | -0.08 |
|  | **P-value** | 0.60 | 0.69 |
| **Caffeine (mg)** | **r** | 0.04 | 0.02 |
|  | **P-value** | 0.87 | 0.89 |
| **quality of life** | **r** | 0.11 | -0.19 |
|  | **P-value** | 0.63 | 0.31 |
| **Insomnia severity index** | **r** | 0.41 | -0.07 |
|  | **P-value** | 0.07 | 0.72 |
| **Subjective happiness** | **r** | 0.34 | -0.03 |
|  | **P-value** | 0.14 | 0.87 |
| **Stress** | **r** | -0.13 | -0.05 |
|  | **P-value** | 0.57 | 0.79 |
| **High-intensity activity (min)** | **r** | . a | -.38* |
|  | **P-value** | - | **>0.04** |
| **Moderate-Intensity Activity (min)** | **r** | 0.07 | -0.29 |
|  | **P-value** | 0.76 | 0.12 |
| **Walking (min)** | **r** | 0.05 | -0.18 |
|  | **P-value** | 0.85 | 0.33 |
| **Sitting time (hr.)** | **r** | -0.15 | -0.08 |
|  | **P-value** | 0.51 | 0.67 |

**Abbreviations: Pearson’s Correlation; r. P-value < 0.05 was considered statistically significant**

***. Correlation is significant at the 0.05 level, **. The correlation is significant at the 0.01 level.**

**a Cannot be computed because at least one of the variables is constant.**

**Supplementary Table 2:** Correlation between bone density variables with age, weight before 10 years/weight in last competition, energy and nutrient intake, questionnaires, BMI, and physical activity among the sample.

|  | | **Non-athletes** | | | | | | | **Retired athletes** | | | | | | |
| --- | --- | --- | --- | --- | --- | --- | --- | --- | --- | --- | --- | --- | --- | --- | --- |
| **Variables** | | **Z- score**  **Neck**  **(Left**  **femur)** | **Z-score**  **Total**  **(Left**  **femur)** | **T-score**  **Neck**  **(Left**  **femur)** | **T-score**  **Total**  **(Left**  **femur)** | **Z-score**  **AP spine** | **T-score**  **AP spine** | **BMC** | **Z- score**  **Neck**  **(Left**  **femur)** | **Z-score**  **Total**  **(Left**  **femur)** | **T-score**  **Neck**  **(Left**  **femur)** | **T-score**  **Total**  **(Left**  **femur)** | **Z-score**  **AP spine** | **T-score**  **AP spine** | **BMC** |
| **Age** | **r** | 0.19 | 0.39 | -0.28 | -0.10 | -0.03 | 0.02 | 0.02 | 0.04 | 0.18 | . ^a^ | . ^a^ | 0.08 | . ^a^ | 0.17 |
|  | **P-value** | 0.46 | 0.13 | 0.71 | 0.89 | 0.88 | 0.97 | 0.90 | 0.80 | 0.33 | - | - | 0.65 | - | 0.36 |
| **Current**  **Weight** | **r** | 0.29 | 0.36 | 0.90 | 0.74 | 0.33 | 0.83 | .59** | 0.33 | .41^*^ | . ^a^ | . ^a^ | 0.00 | . ^a^ | 0.64^**^ |
|  | **P-value** | 0.26 | 0.17 | 0.09 | 0.25 | 0.22 | 0.08 | **>0.01** | 0.08 | 0.03 | - | - | 0.98 |  | **>0.00** |
| **Weight before**  **10 years** | **r** | -0.09 | -0.19 | 0.64 | 0.32 | -0.16 | 0.91* | 0.21 | 0.35 | 0.33 | . ^a^ | . ^a^ | -0.01 | . ^a^ | 0.66^**^ |
|  | **P-value** | 0.75 | 0.49 | 0.36 | 0.68 | 0.57 | **>0.03** | 0.37 | 0.07 | 0.08 | - | - | 0.95 | - | **>0.00** |
| **Fat mass**  **(Kg)** | **r** | 0.33 | 0.44 | 0.59 | 0.85 | 0.21 | 0.60 | 0.31 | 0.23 | 0.36 | . ^a^ | . ^a^ | 0.09 | . ^a^ | 0.39^*^ |
|  | **P-value** | 0.21 | 0.09 | 0.40 | 0.12 | 0.46 | 0.28 | 0.19 | 0.24 | 0.06 | - | - | 0.63 | - | **0.03** |
| **Lean mass**  **(Kg)** | **r** | 0.13 | 0.09 | 0.61 | 0.21 | 0.39 | 0.86 | 0.73** | 0.35 | 0.35 | . ^a^ | . ^a^ | -0.12 | . ^a^ | 0.73^**^ |
|  | **P-value** | 0.63 | 0.73 | 0.39 | 0.79 | 0.14 | 0.06 | **>0.00** | 0.06 | 0.06 | - | - | 0.58 | - | **>0.00** |
| **Calories (kcal)** | **r** | -0.08 | -0.05 | 0.86 | 0.53 | -0.36 | 0.73 | -0.01 | 0.03 | 0.04 | . ^a^ | . ^a^ | -0.18 | . ^a^ | 0.27 |
|  | **P-value** | 0.77 | 0.85 | 0.14 | 0.47 | 0.19 | 0.16 | 0.97 | 0.89 | 0.84 | - | - | 0.35 | - | 0.14 |
| **Protein(g)** | **r** | -0.08 | -0.05 | 0.86 | 0.53 | -0.36 | 0.73 | -0.01 | -0.12 | -0.08 | . a | . a | -0.02 | . a | 0.04 |
|  | **P-value** | 0.77 | 0.85 | 0.14 | 0.47 | 0.19 | 0.16 | 0.97 | 0.53 | 0.68 | - | - | 0.91 | - | 0.83 |
| **CHO(g)** | **r** | -0.24 | -0.24 | 0.94 | 0.81 | -0.01 | 0.36 | -0.08 | 0.33 | 0.38* | . a | . a | -0.04 | . a | 0.44* |
|  | **P-value** | 0.36 | 0.37 | 0.06 | 0.19 | 0.96 | 0.56 | 0.74 | 0.08 | **>0.04** | - | - | 0.98 | - | **>0.01** |
| **Fat (g)** | **r** | 0.15 | 0.22 | 0.49 | 0.09 | -0.35 | 0.71 | 0.02 | -0.29 | -0.37* | . a | . a | -0.33 | . a | -0.07 |
|  | **P-value** | 0.57 | 0.42 | 0.50 | 0.91 | 0.19 | 0.18 | 0.92 | 0.12 | **>0.04** | - | - | 0.08 | - | 0.71 |
| **Saturated Fat (g)** | **r** | 0.17 | 0.19 | 0.93 | 0.67 | -0.04 | 0.53 | -0.01 | -0.12 | -0.20 | . a | . a | -0.11 | . a | -0.04 |
|  | **P-value** | 0.53 | 0.48 | 0.07 | 0.33 | 0.88 | 0.36 | 0.98 | 0.53 | 0.29 | - | - | 0.58 | - | 0.83 |
| **Mono-unsaturated Fat (g)** | r | -0.61^*^ | -0.54^*^ | 0.91 | 0.63 | 0.02 | 0.86 | 0.18 | -0.36 | -0.39* | . a | . a | -0.30 | . a | -0.19 |
|  | **P-value** | **>0.01** | **>0.03** | 0.09 | 0.37 | 0.93 | 0.06 | 0.44 | 0.05 | 0.03 | - | - | 0.11 | - | 0.29 |
| **Poly-unsaturated Fat (g)** | **r** | -0.48 | -0.54^*^ | 0.63 | 0.26 | -0.22 | 0.64 | -0.01 | -0.40* | -0.48** | . a | . a | -0.42* | . a | -0.49** |
|  | **P-value** | 0.058 | **>0.03** | 0.37 | 0.74 | 0.42 | 0.25 | 0.95 | **>0.03** | **>0.01** | - | - | **>0.02** | - | **>0.01** |
| **Trans Fat (g)** | **r** | 0.14 | 0.13 | 0.73 | 0.85 | -0.44 | 0.42 | -0.12 | 0.24 | -0.31 | . a | . a | -0.13 | . a | -0.16 |
|  | **P-value** | 0.60 | 0.63 | 0.27 | 0.15 | 0.10 | 0.48 | 0.59 | 0.20 | 0.09 | - | - | 0.49 |  | 0.41 |
| **Cholesterol (mg)** | **r** | -0.28 | -0.13 | 0.94 | 0.93 | -0.04 | 0.69 | -0.03 | -0.03 | -0.01 | . a | . a | 0.15 | . a | -0.12 |
|  | **P-value** | 0.29 | 0.62 | 0.06 | 0.07 | 0.89 | 0.19 | 0.89 | 0.88 | 0.94 | - | - | 0.44 | - | 0.54 |
| **Omega3 (g)** | **r** | -0.55^*^ | -0.62^*^ | 0.67 | 0.27 | -0.25 | 0.51 | -0.08 | -0.30 | -0.38* | . a | . a | -0.34 | . a | -0.23 |
|  | **P-value** | **>0.03** | **>0.01** | 0.33 | 0.73 | 0.36 | 0.38 | 0.72 | 0.11 | **>0.04** | - | - | 0.07 | - | 0.21 |
| **Omega6 (g)** | **r** | -0.55^*^ | -0.68^**^ | 0.70 | 0.33 | -0.37 | 0.48 | -0.15 | -0.39* | -0.47** | . a | . a | -0.49** | . a | -0.49** |
|  | **P-value** | **>0.02** | **>0.00** | 0.29 | 0.67 | 0.17 | 0.41 | 0.52 | **>0.03** | **>0.01** | - | - | **>0.01** | - | **>0.01** |
| **Vitamin A-RAE (RAE)** | **r** | -0.24 | -0.32 | 0.44 | 0.31 | -0.50 | 0.25 | -0.29 | -0.24 | -0.32 | 0.44 | 0.31 | -0.50 | 0.250 | -0.29 |
|  | **P-value** | 0.37 | 0.22 | 0.55 | 0.69 | 0.05 | 0.68 | 0.20 | 0.37 | 0.22 | 0.55 | 0.69 | 0.05 | 0.685 | 0.20 |
| **Vitamin E-alpha Tocopherol (mg)** | **r** | -0.37 | -0.06 | 0.36 | -0.02 | 0.11 | 0.29 | -0.05 | 0.19 | 0.27 | . ^a^ | . ^a^ | -0.07 | . ^a^ | 0.32 |
|  | **P-value** | 0.15 | 0.81 | 0.64 | 0.98 | 0.71 | 0.64 | 0.83 | 0.30 | 0.16 | - | - | 0.72 | - | 0.09 |
| **Vitamin K (mcg)** | **r** | -0.22 | 0.12 | 0.38 | 0.74 | 0.42 | 0.21 | 0.18 | -0.25 | -0.21 | . ^a^ | . ^a^ | -0.35 | . ^a^ | -0.10 |
|  | **P-value** | 0.42 | 0.66 | 0.62 | 0.26 | 0.12 | 0.74 | 0.45 | 0.19 | 0.27 | - | - | 0.06 | - | 0.58 |
| **Vitamin D-mcg (mcg)** | **r** | -0.42 | -0.41 | 0.86 | 0.78 | -0.28 | 0.55 | -0.10 | -0.02 | -0.04 | . ^a^ | . ^a^ | 0.16 | . ^a^ | -0.16 |
|  | **P-value** | 0.10 | 0.12 | 0.14 | 0.22 | 0.31 | 0.34 | 0.67 | 0.93 | 0.83 | - | - | 0.42 | - | 0.39 |
| **Vitamin B1 (mg)** | **r** | -0.02 | -0.05 | 0.65 | 0.29 | -0.27 | 0.48 | -0.03 | -0.18 | -0.19 | . ^a^ | . ^a^ | -0.26 | . ^a^ | 0.15 |
|  | **P-value** | 0.94 | 0.84 | 0.35 | 0.70 | 0.33 | 0.41 | 0.91 | 0.34 | 0.32 | - | - | 0.18 | - | 0.42 |
| **Vitamin B2 (mg)** | **r** | -0.35 | -0.21 | 0.76 | 0.43 | -0.06 | 0.55 | -0.01 | -0.06 | -0.16 | . ^a^ | . ^a^ | -0.12 | . ^a^ | -0.08 |
|  | **P-value** | 0.19 | 0.44 | 0.24 | 0.57 | 0.82 | 0.34 | 0.97 | 0.75 | 0.40 | - | - | 0.53 | - | 0.67 |
| **Vitamin B3-NE (mg)** | **r** | -0.43 | -0.50^*^ | 0.66 | 0.42 | -0.19 | 0.43 | 0.00 | -0.35 | -0.41^*^ | . ^a^ | . ^a^ | -0.38^*^ | . ^a^ | -0.21 |
|  | **P-value** | 0.09 | **>0.04** | 0.34 | 0.58 | 0.49 | 0.47 | 0.99 | 0.06 | **>0.03** | - | - | **>0.04** | - | 0.27 |
| **Vitamin B6 (mg)** | **r** | -0.40 | -0.33 | 0.79 | 0.51 | 0.01 | 0.43 | -0.06 | 0.04 | -0.08 | . ^a^ | . ^a^ | -0.29 | . ^a^ | 0.09 |
|  | **P-value** | 0.12 | 0.21 | 0.21 | 0.48 | 0.97 | 0.47 | 0.81 | 0.82 | 0.67 | - | - | 0.12 | - | 0.64 |
| **Vitamin B12** | **r** | -0.22 | -0.15 | 0.80 | 0.61 | -0.23 | 0.53 | -0.12 | 0.13 | 0.11 | . ^a^ | . ^a^ | 0.03 | . ^a^ | 0.09 |
|  | **P-value** | 0.40 | 0.57 | 0.19 | 0.39 | 0.39 | 0.36 | 0.60 | 0.51 | 0.56 | - | - | 0.86 | - | 0.61 |
| **Biotin (mcg)** | **r** | 0.09 | 0.11 | 0.94 | 0.69 | -0.16 | 0.63 | 0.03 | -0.07 | -0.08 | . ^a^ | . ^a^ | 0.22 | . ^a^ | -0.04 |
|  | **P-value** | 0.73 | 0.68 | 0.06 | 0.30 | 0.57 | 0.25 | 0.89 | 0.72 | 0.67 | - | - | 0.25 | - | 0.83 |
| **Vitamin C (mg)** | **r** | -0.07 | -0.17 | -0.12 | 0.33 | 0.09 | -0.63 | -0.28 | 0.26 | 0.20 | . ^a^ | . ^a^ | -0.11 | . ^a^ | 0.23 |
|  | **P-value** | 0.78 | 0.52 | 0.88 | 0.67 | 0.74 | 0.25 | 0.24 | 0.18 | 0.29 | - | - | 0.56 | - | 0.23 |
| **Folate, DFE (mcg)** | **r** | -0.02 | 0.13 | 0.47 | 0.29 | 0.09 | 0.19 | 0.11 | -0.14 | -0.08 | . ^a^ | . ^a^ | -0.06 | . ^a^ | 0.25 |
|  | **P-value** | 0.95 | 0.62 | 0.53 | 0.71 | 0.74 | 0.76 | 0.63 | 0.46 | 0.68 | - | - | 0.74 | - | 0.18 |
| **Pantothenic acid (mg)** | **r** | -0.38 | -0.31 | 0.68 | 0.44 | -0.14 | 0.46 | -0.01 | -0.26 | -0.33 | . ^a^ | . ^a^ | -0.15 | . ^a^ | -0.19 |
|  | **P-value** | 0.15 | 0.24 | 0.32 | 0.56 | 0.61 | 0.44 | 0.98 | 0.18 | 0.08 | - | - | 0.43 | - | 0.29 |
| **Calcium (mg)** | **r** | -0.18 | -0.19 | 0.68 | 0.42 | -0.09 | 0.51 | 0.11 | 0.31 | 0.20 | . ^a^ | . ^a^ | 0.12 | . ^a^ | 0.13 |
|  | **P-value** | 0.51 | 0.48 | 0.32 | 0.58 | 0.75 | 0.38 | 0.65 | 0.09 | 0.29 | - | - | 0.53 | - | 0.49 |
| **Chromium (mcg)** | **r** | 0.02 | 0.04 | 0.57 | 0.28 | 0.12 | 0.39 | 0.07 | -0.01 | -0.051 | . ^a^ | . ^a^ | 0.09 | . ^a^ | 0.33 |
|  | **P-value** | 0.95 | 0.89 | 0.43 | 0.72 | 0.68 | 0.51 | 0.78 | 0.96 | 0.79 | - | - | 0.66 | - | 0.08 |
| **Copper (mg)** | **r** | -0.21 | -0.19 | 0.70 | 0.34 | -0.39 | 0.56 | -0.21 | 0.12 | 0.18 | . ^a^ | . ^a^ | 0.02 | . ^a^ | 0.13 |
|  | **P-value** | 0.44 | 0.47 | 0.29 | 0.65 | 0.15 | 0.32 | 0.37 | 0.54 | 0.36 | - | - | 0.92 | - | 0.49 |
| **Iodine (mcg)** | **r** | -0.11 | -0.23 | 0.77 | 0.58 | -0.19 | 0.58 | 0.09 | 0.08 | 0.02 | . ^a^ | . ^a^ | 0.29 | . ^a^ | 0.07 |
|  | **P-value** | 0.67 | 0.40 | 0.23 | 0.42 | 0.48 | 0.31 | 0.68 | 0.67 | 0.91 | - | - | 0.12 | - | 0.69 |
| **Iron (mg)** | **r** | -0.17 | -0.01 | 0.94 | 0.68 | -0.08 | 0.70 | -0.06 | -0.18 | -0.15 | . ^a^ | . ^a^ | -0.11 | . ^a^ | 0.10 |
|  | **P-value** | 0.54 | 0.97 | 0.06 | 0.31 | 0.77 | 0.18 | 0.79 | 0.34 | 0.43 | - | - | 0.55 | - | 0.6 |
| **Magnesium (mg)** | **r** | -0.19 | -0.05 | 0.64 | 0.25 | 0.20 | 0.59 | 0.17 | -0.06 | -0.04 | . ^a^ | . ^a^ | -0.16 | . ^a^ | 0.22 |
|  | **P-value** | 0.49 | 0.84 | 0.36 | 0.74 | 0.46 | 0.29 | 0.47 | 0.74 | 0.83 | - | - | 0.40 | - | 0.24 |
| **Manganese (mg)** | **r** | -0.02 | 0.04 | 0.61 | 0.25 | 0.01 | 0.54 | 0.06 | 0.16 | 0.22 | . ^a^ | . ^a^ | -0.06 | . ^a^ | 0.39^*^ |
|  | **P-value** | 0.95 | 0.87 | 0.39 | 0.74 | 0.97 | 0.35 | 0.79 | 0.41 | 0.25 | - | - | 0.74 | - | 0.03 |
| **Molybdenum (mcg)** | **r** | -0.16 | -0.16 | 0.95^*^ | 0.95^*^ | -0.16 | 0.52 | -0.07 | 0.13 | 0.13 | . ^a^ | . ^a^ | -0.13 | . ^a^ | 0.14 |
|  | **P-value** | 0.54 | 0.55 | **>0.04** | **>0.04** | 0.56 | 0.36 | 0.76 | 0.52 | 0.51 | - | - | 0.51 | - | 0.48 |
| **Phosphorus (mg)** | **r** | -0.31 | -0.32 | 0.75 | 0.40 | -0.13 | 0.56 | 0.05 | -0.07 | -0.15 | . ^a^ | . ^a^ | -0.14 | . ^a^ | 0.10 |
|  | **P-value** | 0.25 | 0.22 | 0.25 | 0.59 | 0.64 | 0.33 | 0.84 | 0.72 | 0.44 | - | - | 0.47 | - | 0.59 |
| **Potassium (mg)** | **r** | -0.38 | -0.30 | 0.80 | 0.47 | 0.14 | 0.62 | 0.09 | 0.17 | 0.08 | . ^a^ | . ^a^ | -0.17 | . ^a^ | 0.29 |
|  | **P-value** | 0.14 | 0.26 | 0.19 | 0.53 | 0.62 | 0.26 | 0.71 | 0.39 | 0.68 | - | - | 0.39 | = | 0.12 |
| **Selenium (mcg)** | **r** | -0.36 | -0.38 | 0.80 | 0.59 | -0.01 | 0.47 | 0.06 | -0.24 | -0.25 | . ^a^ | . ^a^ | -0.17 | . ^a^ | -0.09 |
|  | **P-value** | 0.17 | 0.14 | 0.19 | 0.40 | 0.96 | 0.42 | 0.79 | 0.21 | 0.19 | - | - | 0.38 | - | 0.61 |
| **Sodium (mg)** | **r** | -0.03 | 0.09 | 0.05 | -0.26 | -0.26 | 0.23 | -0.24 | 0.24 | 0.28 | . ^a^ | . ^a^ | 0.19 | . ^a^ | 0.27 |
|  | **P-value** | 0.91 | 0.74 | 0.94 | 0.74 | 0.36 | 0.71 | 0.30 | 0.21 | 0.14 | - | - | 0.33 | - | 0.15 |
| **Zinc (mg)** | **r** | -0.27 | -0.24 | 0.89 | 0.59 | 0.07 | 0.62 | 0.08 | -0.06 | -0.17 | . ^a^ | . ^a^ | -0.04 | . ^a^ | 0.17 |
|  | **P-value** | 0.31 | 0.37 | 0.11 | 0.41 | 0.80 | 0.26 | 0.75 | 0.76 | 0.37 | - | - | 0.85 | - | 0.37 |
| **Caffeine (mg)** | **r** | 0.16 | 0.24 | -0.99^*^ | -0.81 | -0.10 | -0.96^**^ | -0.11 | -0.08 | -0.02 | . ^a^ | . ^a^ | -0.05 | . ^a^ | 0.04 |
|  | **P-value** | 0.55 | 0.37 | **>0.01** | 0.19 | 0.715 | **>0.01** | 0.63 | 0.66 | 0.92 | - | - | 0.81 | - | 0.82 |
| **Quality of life** | **r** | 0.31 | 0.30 | -0.69 | -0.63 | 0.18 | -0.71 | -0.08 | 0.11 | 0.11 | . ^a^ | . ^a^ | 0.32 | . ^a^ | 0.04 |
|  | **P-value** | 0.25 | 0.26 | 0.31 | 0.37 | 0.51 | 0.18 | 0.72 | 0.58 | 0.57 | - | - | 0.09 | - | 0.84 |
| **Insomnia severity index** | **r** | 0.07 | 0.18 | 0.88 | 0.92 | 0.08 | 0.83 | 0.17 | -0.15 | -0.21 | . ^a^ | . ^a^ | -0.13 | . ^a^ | -0.30 |
|  | **P-value** | 0.79 | 0.51 | 0.11 | 0.07 | 0.772 | 0.08 | 0.48 | 0.43 | 0.26 | - | - | 0.51 | - | 0.11 |
| **Subjective happiness** | **r** | 0.19 | 0.42 | -0.69 | -0.69 | 0.44 | 0.14 | 0.20 | 0.19 | 0.11 | . ^a^ | . ^a^ | -0.09 | . ^a^ | -0.07 |
|  | **P-value** | 0.46 | 0.11 | 0.31 | 0.31 | 0.09 | 0.82 | 0.39 | 0.33 | 0.55 | - | - | 0.66 | - | 0.71 |
| **Perceived Stress** | **r** | 0.29 | 0.13 | -0.42 | -0.11 | -0.17 | -0.70 | -0.35 | 0.44^*^ | 0.45^*^ | . ^a^ | . ^a^ | 0.49^**^ | . ^a^ | 0.12 |
|  | **P-value** | 0.27 | 0.63 | 0.58 | 0.89 | 0.54 | 0.18 | 0.13 | **>0.02** | **>0.01** | - | - | **>0.01** | - | 0.54 |
| **BMI** | **r** | 0.31 | 0.37 | 0.83 | 0.99^**^ | 0.04 | 0.71 | 0.26 | 0.27 | 0.37 | . ^a^ | . ^a^ | 0.01 | . ^a^ | 0.42^*^ |
|  | **P-value** | 0.25 | 0.16 | 0.17 | **>0.01** | 0.89 | 0.18 | 0.26 | 0.15 | 0.051 | - | - | 0.96 |  | 0.02 |
| **High-intensity activity**  **(min)** | **r** | . ^a^ | . ^a^ | . ^a^ | . ^a^ | . ^a^ | . ^a^ | . ^a^ | 0.12 | 0.03 | . a | . a | 0.18 | . a | -0.18 |
|  | **P-value** | - | - | - | - | - | - | - | 0.52 | 0.87 | - | - | 0.35 | - | 0.34 |
| **Moderate-Intensity**  **Activity**  **(min)** | **r** | 0.18 | -0.03 | . ^a^ | . ^a^ | 0.01 | 0.58 | 0.10 | 0.15 | 0.05 | . a | . a | 0.18 | . a | -0.25 |
|  | **P-value** | 0.51 | 0.90 | - | - | 0.97 | 0.30 | 0.67 | 0.43 | 0.81 | - | - | 0.39 | - | 0.17 |
| **Walking**  **(min)** | **r** | 0.20 | 0.29 | 0.80 | .98^*^ | -0.02 | -0.22 | 0.39 | 0.21 | 0.08 | . a | . a | 0.10 | . a | -0.12 |
|  | **P-value** | 0.48 | 0.30 | 0.19 | **> 0.01** | 0.95 | 0.72 | 0.11 | 0.28 | 0.69 | - | - | 0.59 | - | 0.51 |
| **Sitting time (hr.)** | **r** | -0.14 | -0.21 | -0.59 | -0.17 | -0.38 | 0.58 | -0.33 | -0.09 | -0.13 | . a | . a | 0.15 | . a | 0.06 |
|  | **P-value** | 0.61 | 0.44 | 0.41 | 0.83 | 0.16 | 0.30 | 0.15 | 0.63 | 0.50 | - | - | 0.44 | - | 0.73 |

**Abbreviations: Pearson’s Correlation; r. CHO: Carbohydrate. P-value < 0.05 was considered statistically significant*. Correlation is significant at the 0.05 level, **. The correlation is significant at the 0.01 level.** ^a^ **Cannot be computed because at least one of the variables is constant**
